# Fanconi anemia pathway regulation by FANCI in prostate cancer

**DOI:** 10.3389/fonc.2023.1260826

**Published:** 2023-10-30

**Authors:** Heidi Kaljunen, Sinja Taavitsainen, Roosa Kaarijärvi, Eerika Takala, Ville Paakinaho, Matti Nykter, G. Steven Bova, Kirsi Ketola

**Affiliations:** ^1^ Institute of Biomedicine, University of Eastern Finland, Kuopio, Finland; ^2^ Faculty of Medicine and Health Technology, Tampere University and Tays Cancer Center, Tampere, Finland

**Keywords:** prostate, cancer, treatment resistance, DNA damage, carboplatin

## Abstract

Prostate cancer is one of the leading causes of death among men worldwide, and thus, research on the genetic factors enabling the formation of treatment-resistant cancer cells is crucial for improving patient outcomes. Here, we report a cell line-specific dependence on *FANCI* and related signaling pathways to counteract the effects of DNA-damaging chemotherapy in prostate cancer. Our results reveal that *FANCI* depletion results in significant downregulation of Fanconi anemia (FA) pathway members in prostate cancer cells, indicating that *FANCI* is an important regulator of the FA pathway. Furthermore, we found that FANCI silencing reduces proliferation in p53-expressing prostate cancer cells. This extends the evidence that inactivation of *FANCI* may convert cancer cells from a resistant state to an eradicable state under the stress of DNA-damaging chemotherapy. Our results also indicate that high expression of FA pathway genes correlates with poorer survival in prostate cancer patients. Moreover, genomic alterations of FA pathway members are prevalent in prostate adenocarcinoma patients; mutation and copy number information for the FA pathway genes in seven patient cohorts (N = 1,732 total tumor samples) reveals that 1,025 (59.2%) tumor samples have an alteration in at least one of the FA pathway genes, suggesting that genomic alteration of the pathway is a prominent feature in patients with the disease.

## Introduction

Several anticancer chemotherapies work by producing DNA damage, leading to cell death. As a treatment resistance mechanism to cell cycle arrest-causing DNA-damaging agents, DNA repair pathways take part in repairing DNA and promoting the survival of cancer cells during chemotherapy. To date, several DNA repair inhibitors have been combined with conventional chemotherapy drugs to improve cancer cell-specific DNA damage, cell cycle arrest, and cell death.

FANCI is a member of the Fanconi anemia (FA) protein family, which is involved in sensing and repairing DNA damage as part of DNA damage response (DDR) and especially in regulation of interstrand cross-link (ICL) repair, to prevent genomic instability and replication stress (1–4). FANCI protein associates with another FA protein, FANCD2, forming a so-called ID complex, which is activated upon DNA damage recognition through monoubiquitination of both proteins by the FA core complex (1, 2, 5). The ID complex is then loaded onto the chromatin to recruit other DNA repair proteins and thus facilitate DNA repair. In addition to the canonical function of the FA pathway in the repair of ICL-type DNA damage, this pathway has been associated with the repair of dsDNA breaks and UV-induced lesions, as well as with the regulation of DNA replication forks (6–9). FA itself is a clinically and genetically heterogeneous disorder that is characterized in part by developmental abnormalities and a high predisposition to various cancers arising from germline mutation in one of at least 21 FA genes. Currently, over 20 genes are known to be involved in the FA pathway, including FANCA, FANCB, FANCC, FANCD1 (BRCA2), FANCD2, FANCE, FANCF, FANCG (XRCC9), FANCI, FANCJ (BRIP1 or BACH1), FANCL, FANCM, FANCN (PALB2), FANCO (RAD51C), FANCP (SLX4), FANCQ (ERCC4), FANCR (RAD51), FANCS (BRCA1), FANCT (UBE2T), FANCU (XRCC2), FANCV (REV7), and FANCW (alternative gene names in parenthesis) (10, 11). Additionally, several other proteins are involved in the regulation of FA pathway activity, including two FA-associated proteins (FAAP100 and FAAP24) and the DNA damage checkpoint kinase ataxia telangiectasia and Rad3-related (ATR), which regulates the activation of the FA pathway through phosphorylation of FANCI/D2 (5, 12–15). In particular, phosphorylation of FANCI provides an on/off switch for the FA pathway activation, as it is required for the monoubiquitination of both FANCI and FANCD2 and also provides a protective mechanism toward the action of the deubiquitinating enzyme USP1:UAF1 (5, 15, 16).

Though individuals with FA form the basis for understanding the importance of the FA pathway in maintaining genomic integrity, it has been recently recognized that FA pathway-related genes also contribute to cancer susceptibility in individuals without FA (17). Several germline mutations and variants of FA pathway genes have been reported as risk factors in cancer in non-FA patients/individuals; namely, mutations in *BRCA1* (*FANCS*), *PALB2* (*FANCN*), and *BRCA2* (*FANCD1*) have been shown to lead to increased risk of breast and ovarian cancers, as well as prostate cancer (18–20). Similarly, association with increased cancer susceptibility has been associated with germline variants of *FANCA*, *FANCM*, and *FANCR* (*RAD51*), among others (21–25). To this end, the available data from a recent pan-cancer whole-genome sequencing analysis of solid metastatic tumors indicates that roughly 20% of germline drivers are involved in the FA pathway, thus implying that germline variants of FA genes are important factors in cancer predisposition also in non-FA patients (25).

We recently reported FANCI as a candidate DNA repair-related target for converting metastatic prostate cancer tumor subclones from resistant to eradicable (26). Prostate cancer tumor cells harboring a heterozygous deletion of *FANCI* were selectively eradicated by chemotherapy, while those with intact *FANCI* resisted chemotherapy, suggesting that FANCI could be a possible therapeutic target to convert resistant cancer cells to an eradicable state. Here, we studied the role of the whole FA pathway in prostate cancer and in particular the role of FANCI in cancer cell resistance to chemotherapy through regulation of cell cycle and DNA repair by the FA pathway. Our results indicate that FANCI silencing induces cell cycle arrest through the downregulation of DNA replication/synthesis and cell cycle checkpoint genes. Moreover, we found that FANCI silencing significantly reduced the proliferation of prostate cancer cells expressing functional p53 tumor suppressor protein and potentiated the growth inhibitory effect of carboplatin in these cells. Furthermore, silencing of FANCI resulted in significant downregulation of several FA complex members both at mRNA and protein levels, indicating the potential regulatory function of FANCI on the FA pathway in prostate cancer cells.

## Results

### FANCI depletion induces p21-dependent cell cycle arrest in LNCaP cells

**Figure 1:**
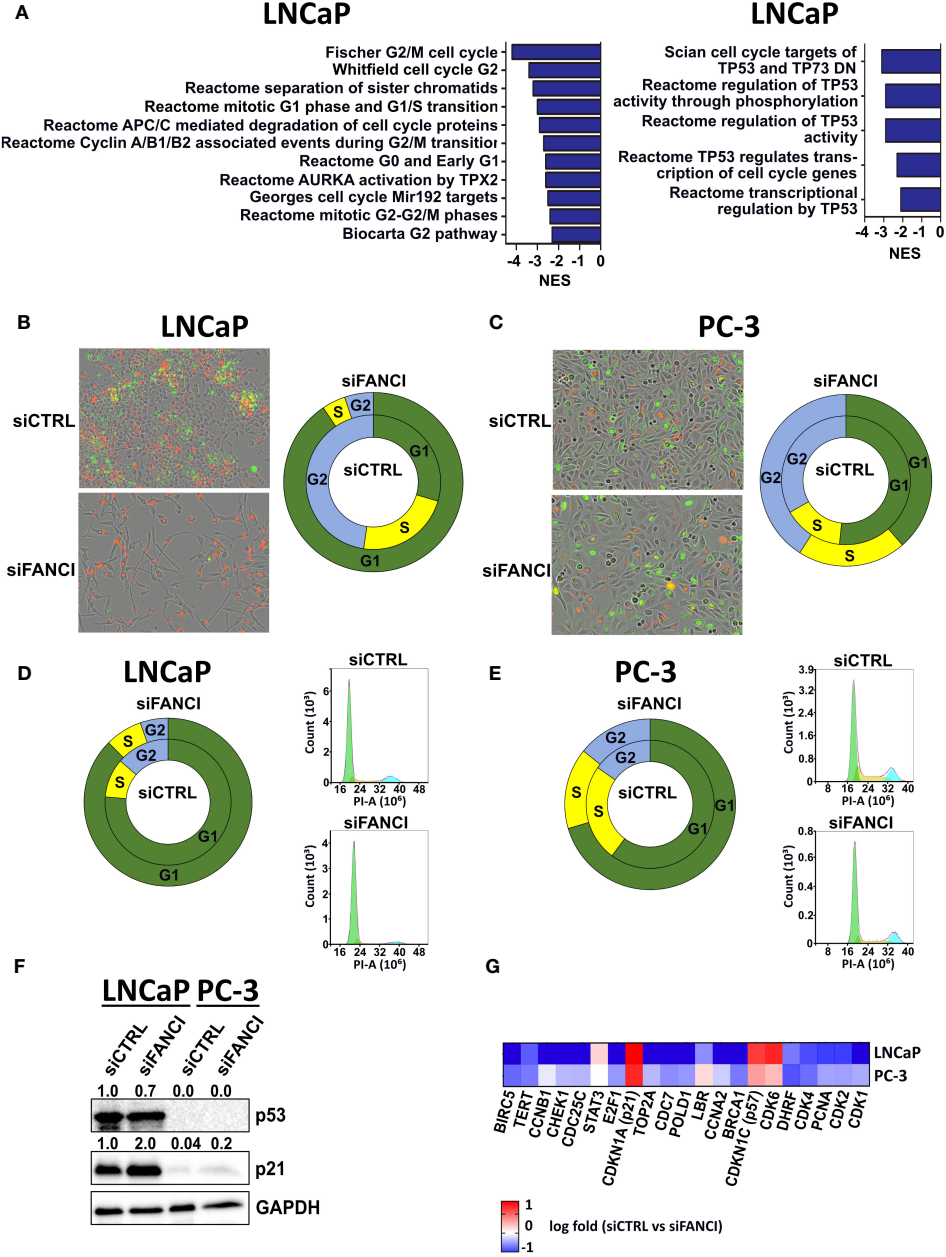
FANCI silencing induces cell cycle arrest in LNCaP cells. **(A)** Left: gene sets related to cell cycle regulation that were downregulated in LNCaP cells when compared to PC-3 cells in response to FANCI depletion based on RNA-sequencing and Gene Set Enrichment Analysis (GSEA). Right: p53 (*TP53*) regulation-related gene sets in LNCaP siFANCI GSEA. N = 3, normalized enrichment score (NES) <−2, and p-value <0.05. **(B)** FANCI-silenced (siFANCI) LNCaP cells undergo G1 arrest, while control cells (siCTRL) continue dividing normally based on IncuCyte live cell imaging of LNCaP cells with fluorescence-labeled cell cycle markers Cdt1 (red, G1 phase cells) and geminin (green, G2 phase cells). The S phase cells have both Cdt1 and geminin, thus appearing yellow. Left: IncuCyte images of the siCTRL and siFANCI LNCaP cells. Right: donut plot showing percentages of cells in G1 (green section), S (yellow section), and G2 (blue section) phases based on analysis with IncuCyte software (inner circle, siCTRL; outer circle, siFANCI). **(C)** In similarly labeled PC-3 cells, no significant difference between siCTRL and siFANCI cells is observed. Left: IncuCyte images of the siCTRL and siFANCI PC-3 cells. Right: donut plot with the calculated percentages of cells in G1, S, and G2 phases based on the IncuCyte analysis (inner circle, siCTRL; outer circle, siFANCI). **(D)** Flow cytometry analysis on FANCI-silenced and control siRNA LNCaP cells correlate with the IncuCyte live cell imaging data showing increased percentage of cells in G1 phase in response to FANCI silencing. Right: donut plot showing percentages of cells in G1, S, and G2 phases (inner circle, siCTRL; outer circle, siFANCI). Left: flow cytometry histograms of cell cycle assay by propidium iodide (PI) method created using NovoExpress software. **(E)** In the flow cytometry analysis of siFANCI PC-3 cells, no marked difference is seen when compared to the control. Right: donut plot representing the percentages of G1, S, and G2 phases (inner circle, siCTRL; outer circle, siFANCI). Left: flow cytometry histograms of PI-based cell cycle analysis using NovoExpress software. **(F)** Immunoblotting analysis of p53 and p21, known cell cycle regulators, in FANCI-silenced (siFANCI) or control siRNA (siCTRL) LNCaP and PC-3 cells showing a lack of p53 expression in PC-3 cells and p21 upregulation in LNCaP cells in response to FANCI silencing. GAPDH was used as reference. The numeral values above the blots refer to relative band intensities for p53 and p21 normalized to GAPDH. White space was used to make explicit for the grouping of blots cropped from different gels. **(G)** Heatmap based on the RNA-seq data from FANCI-silenced LNCaP and PC-3 cells showing the differential expression of several central cell cycle arrest-associated genes between the two cell lines.

We recently reported that prostate cancer tumor cells harboring heterozygous deletion of *FANCI* were selectively eradicated by chemotherapy, while those with intact *FANCI* resisted chemotherapy, suggesting that FANCI could be a possible therapeutic target to convert resistant cancer cells to eradicable states (26). Our preliminary results also suggested that there are cell line-specific effects of FANCI depletion in prostate cancer cells since LNCaP cells slowed their proliferation rate in response to FANCI silencing while PC-3 cells grew normally. Thus, we initially hypothesized that FANCI may be differently expressed in LNCaP and PC-3 prostate cancer cell lines. To explore which prostate cancer cell line models could be utilized to model the resistant and eradicated states, we determined the FANCI mRNA and protein expression levels in LNCaP, PC-3, 22Rv1, VCaP, and DU-145 prostate cancer cells. The results revealed that differences in FANCI expression do not explain the differential responses on FANCI depletion in PC-3 and LNCaP cells, as there are no statistically significant differences in FANCI expression between LNCaP, PC-3, 22Rv1, VCaP, and DU-145 prostate cancer cells at mRNA and protein levels (**Supplementary Figure S1**). To determine other explanations for the differential responses in PC-3 and LNCaP cells on FANCI depletion, we performed RNA-sequencing on FANCI-silenced LNCaP and PC-3 cells to analyze the alterations compared to siRNA controls. The enriched curated gene sets in response to FANCI silencing were analyzed using Gene Set Enrichment Analysis (GSEA) and the Molecular Signatures Database (MSigDB) (27–29). Overall, the number of both up- and downregulated genes (RNA-seq data, log fold above 0.5 or below−0.5 with adjusted p-value <0.05) in LNCaP cells in response to FANCI silencing was significantly higher than in PC-3 cells (**Supplementary Figure S2A**), indicating that FANCI has an important regulatory role in LNCaP cells. This is supported by the finding that the number of downregulated gene sets in GSEA was also higher in LNCaP cells than in PC-3 cells (**Supplementary Figure S2B**). Closer investigation of the gene sets exclusively downregulated in FANCI-silenced LNCaP cells revealed that several of these sets were pathways/processes related to DNA damage response, mainly through downregulation of FA pathway members, but interestingly, many were related to the regulation of cell cycle (**Supplementary Table 2**, **Figure 1A**). In particular, gene sets including targets and regulators of p53, an important tumor suppressor and apoptosis and cell cycle arrest regulator (30–32), were exclusively downregulated in LNCaP cells (**Figure 1A**). These results suggest that the reduced proliferation associated with FANCI depletion in LNCaP cells could be a result of cells entering apoptosis or cell cycle arrest and the associated accumulation of DNA damage. As LNCaP cells possess wild-type TP53 gene while PC-3 have TP53 truncating mutations and thus do not express p53 (p53-null) (33), p53 status might play a role in separating FANCI-responsive and non-responsive cell lines from one another. In summary, these RNA-seq results on LNCaP and PC-3 cells suggest that FANCI is a potential regulatory factor for biological pathways related to DNA replication/DNA repair and cell cycle specifically in LNCaP cells, and this effect could be p53 dependent.

The observed downregulation of gene sets related to cell cycle and p53 regulation in response to FANCI silencing in LNCaP cells led us to investigate whether the reduced growth rate of LNCaP cells is a result of cell cycle arrest and how this links to the cell cycle regulator and known tumor suppressor p53. To this end, we first created LNCaP, 22Rv1, and PC-3 cell cycle cell lines using IncuCyte Cell Cycle Green/Red lentivirus reagent, which allowed real-time monitoring of the G1 and G2/S phases through fluorescence-labeled Cdt1 and Geminin, known regulators of cell cycle progression. We included the 22Rv1 prostate cancer cell line as this cell line showed the highest FANCI protein expression (**Supplementary Figure 1**) and in terms of *TP53* gene functional status falls between LNCaP and PC-3 cells as 22Rv1 cells harbor a monoallelic *TP53* mutation (WT/Q331R), resulting in a relatively low p53 protein expression (33). Interestingly, we found that almost all FANCI-silenced LNCaP cells were arrested in the G1 phase 4 days post-siRNA transfection (red cells; calculated fractions of cells in the G1 phase 91%, in the S phase 4%, and in the G2 phase 5%), while siCTRL control cells’ cell cycle progression continued normally (calculated percentages of cells in the G1 phase 30%, in the S phase 23%, and in the G2 phase 47%) (**Figure 1B**). No difference in cell cycle response was observed between siRNA control and FANCI-silenced cells in PC-3 cells and rather surprisingly neither in 22Rv1 cells (**Figure 1C**, **Supplementary Figure 3A**). However, FANCI silencing did appear to affect the growth rate of 22Rv1 cells based on IncuCyte cell confluency analysis (**Supplementary Figure S3B**). To verify the observed difference specifically between LNCaP and PC-3 cells, we also performed flow cytometry analysis on both LNCaP and PC-3 cells exposed to FANCI siRNA and stained with propidium iodide (PI). Cells transfected with non-targeting siRNA (siCTRL) were used as controls. As the FANCI silencing effect on G1 arrest was seen already on day 4 based on our IncuCyte analysis, this timepoint was chosen for the flow cytometry analysis. As expected, also according to the flow cytometry data, a significantly larger fraction of the FANCI-depleted LNCaP cells were in the G1 phase than in the S or G2 phase (85% of analyzed cells in the G1 phase, 10% in the S phase, and 5% in the G2 phase; **Figure 1D**) when compared to control siRNA cells (71% of analyzed cells in the G1 phase, 17% in the S phase, and 12% in the G2 phase; **Figure 1D**), thus supporting the live cell imaging results. Furthermore, the flow cytometry analysis on PC-3 cells also coincided with the live cell imaging data, as only a modest response to FANCI silencing in comparison to control siRNA was observed (**Figure 1E**).

Next, we wanted to investigate the role of p53 in the FANCI depletion-induced cell cycle effects. As LNCaP cells have wild-type p53 and PC-3 cells fs-sc/del p53 genotype as mentioned earlier, we hypothesized that the difference in p53 status could explain the observed difference in response to FANCI silencing in LNCaP and PC-3 cells. Our GSEA data indicated that a set of p53 target genes encoding important cell cycle regulators from cyclin and cyclin kinase family (such as CCNB1 and CDK2) and cell division cycle 25 (CDC25) phosphatases (CDC25A, CDC25B, and CDC25C) (34, 35) was exclusively downregulated in LNCaP cells after FANCI depletion in comparison to PC-3 (**Supplementary Table 2**, **Figure 1A**). Thus, we first studied if FANCI depletion affects p53 protein expression in LNCaP cells and also wanted to confirm its absence in PC-3 cells using immunoblotting. 22Rv1 cells were also included in the analysis, as they have one wild-type copy of *TP53* gene but, unlike LNCaP cells, did not undergo G1 arrest based on our IncuCyte live cell imaging analysis. As p53 is known to require a cyclin-dependent kinase (CDK) inhibitor p21 (also known as p21WAF1/Cip1) for the transcriptional repression of cell cycle genes during G1 cell cycle arrest (36–38), we also analyzed the p21 protein expression in LNCaP, 22Rv1, and PC-3 cells. The results showed that, indeed, both LNCaP and 22Rv1 cells express p53, while no p53 expression was observed in PC-3 cells (**Figure 1F**, **Supplementary Figure S3C**). Surprisingly, in LNCaP cells, FANCI silencing did not appear to affect markedly p53 protein levels, yet p21 was upregulated in response to FANCI depletion (**Figure 1F**), indicating p21-mediated induction of G1 arrest. Interestingly, though PC-3 cells showed overall very weak expression of p21, there was an increase in p21 level in FANCI siRNA exposed cells in comparison to control cells (**Figure 1F**). However, as the G1/S checkpoint is p53-dependent, cells with p53 deletion no longer arrest at G1/S transition (39–41), which supports our observation that p53 null PC-3 cells do not undergo G1 arrest upon FANCI depletion. Interestingly, in 22Rv1 cells, FANCI silencing resulted in a slight reduction of expression of p53 in comparison to control siRNA cells, while no apparent difference in p21 levels was observed (siFANCI vs. siCTRL) (**Supplementary Figure 3C**). This could indicate that in 22Rv1 cells, the observed FANCI silencing reduced growth is not a result of p53-mediated cell cycle arrest, but further studies would be required to gain insights into the underlying mechanisms. Overall, 22Rv1 cells appear to represent an interesting intermediate between LNCaP and PC-3 cells in response to FANCI depletion.

We then examined our RNA-sequencing data in more detail, focusing on the known cell cycle regulators and targets of p53/p21 that are specifically involved in G1/S checkpoint and G1 arrest. We were especially interested in genes differentially expressed in LNCaP cells in comparison to PC-3 cells in response to FANCI silencing. First, we found that the expression of cyclin-dependent kinases CDK1 and CDK2 and proliferating cell nuclear antigen (PCNA) was over twofold lower in LNCaP cells than in PC-3 cells in response to FANCI silencing (log fold in LNCaP vs. PC-3: CDK1 −1.30 vs. −0.30, CDK2 −0.99 vs. −042, and PCNA −1.00 vs. −0.41) (**Figure 1G**). CDK1 and CDK2 are both key regulators in transitions from the G1/S phase to the S phase, and their downregulation is associated with p53/p21-dependent cell cycle arrest, while PCNA is required for DNA synthesis and progression to the S phase and is directly bound and inhibited by p21 on protein level (38). The induction of p53/p21-dependent cell cycle arrest in response to FANCI depletion was further supported by our finding that several genes negatively regulated by p53, including Cyclin A2 (CCNA2) and CDC7 kinase (37, 42), were downregulated in LNCaP cells (**Figure 1G**). Furthermore, the upregulation of both p21 (*CDKN1A*) and p57 (*CDKN1C*) was also observed in RNA-seq data showing log fold changes of 1.7 and 1.3, respectively, in LNCaP cells (siCTRL vs. siFANCI) (**Figure 1G**). Like with p21, the upregulation of p57 is also associated with G1 arrest (43). Interestingly, also, a number of p21 target genes that are downregulated upon G1 arrest (38) were also downregulated in LNCaP cells in response to FANCI silencing (**Figure 1G**). These include transcription factor E2F1, which is directly repressed by p21, thus also repressing transcriptional activity of E2F1 resulting in inhibition of cell cycle progression (38, 44). In summary, these results indicate that FANCI depletion induces cell cycle arrest in prostate cancer cells with wild-type p53.

### Depletion of FANCI markedly attenuates the accumulation of FA core complex members

**Figure 2:**
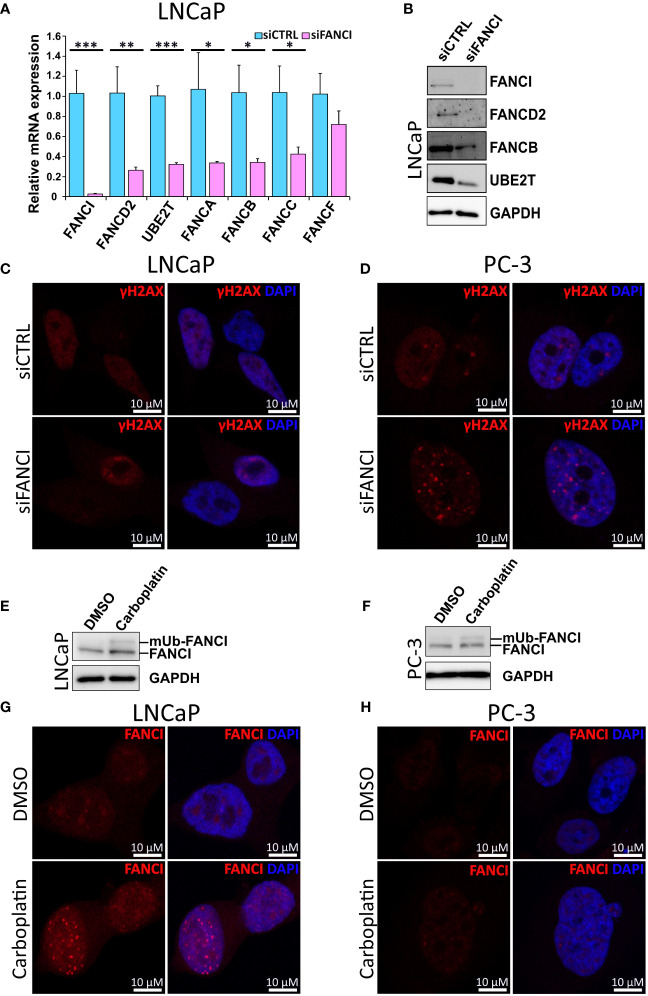
Silencing of FANCI in cell lines with functional Fanconi Anemia pathway results in reduced expression of other FA complex components. **(A)** The effect of FANCI silencing on other key FA complex members, namely FANCA, FANCB, FANCC, FANCD2 and UBE2T (FANCT) on mRNA level in LNCaP cells analyzed using RT-qPCR. Bars represent mean±SD with n=3. P-values shown as asterisks (*p ≤ 0.05, **p ≤ 0.01 and ***p ≤ 0.001). **(B)** The effect of FANCI silencing on FANCI, FANCB, UBE2T and FANCD2 protein levels in LNCaP cells analyzed using immunoblotting. GAPDH antibody was used as a housekeeping control. White space was used to make explicit for the grouping of blots cropped from different gels. **(C)** Immunofluorescence images of LNCaP cells with either FANCI or control siRNA stained with γH2AX (red), which is a known marker for DNA damage. DAPI staining (blue) was used to visualize the nuclei of the cells. **(D)** Results of the immunofluorescence imaging of FANCI or control siRNA PC-3 cells stained with γH2AX (red) and nuclei DNA binding DAPI (blue). **(E)** The effect of Carboplatin (5 µM) on FANCI monoubiquitination in LNCaP prostate cancer cell line analyzed using Western blot of FANCI (140 kDa) and monoubiquitinated FANCI (150 kDa) in comparison to DMSO control cells. Upon Carboplatin exposure, a second band at about 150 kDa corresponding to monoubiquitinated FANCI is observed. White space was utilized to highlight the grouping of blots cropped from different gels. **(F)** Similarly, also in PC-3 cells exposure to Carboplatin results in monoubiquitination of FANCI based on Western blot analysis. **(G)** Immunofluorescence analysis of LNCaP cells exposed to either DMSO (control, ctrl) or Carboplatin (5 µM) using antibody against FANCI (red) to determine the localization and overall expression of FANCI. The formation of distinct nuclear foci in response to Carboplatin indicates activation of FANCI and FA pathway. **(H)** Immunofluorescence analysis of PC-3 cells exposed to DMSO or Carboplatin using FANCI antibody (red). Unlike in LNCaP cells, in PC-3 cells only faint FANCI signal was observed. In both LNCaP and PC-3 cells DAPI staining (blue) was used to visualize nuclear DNA.

Next, we further explored whether the observed cell cycle arrest is a result of inhibition of both FANCI and the associated components of the FA DNA damage repair pathway. First, we analyzed the mRNA expression levels of FA complex members required for functional FA pathway-dependent DNA repair in LNCaP cells exposed to either FANCI or control siRNA using both qPCR and our RNA-sequencing data. Interestingly, we found that silencing of FANCI effectively downregulated not only the mRNA expression of FANCD2, the heterodimeric partner of FANCI, but also necessary subcomplexes of the FA core complex (**Figure 2A**, **Supplementary Figure S4A**). Furthermore, the expression of UBE2T (FANCT), which is needed for the ubiquitination of FANCI and which we have also shown to be upregulated in prostate cancer cells resistant to the anti-androgen therapy drug enzalutamide (45), was significantly reduced (**Figure 2A**, **Supplementary Figure S4A**). The same phenomenon was also observed on FANCD2, FANCB, and UBE2T protein levels analyzed using immunoblotting (**Figure 2B**). To find out if these findings also applied to other prostate cancer cell lines and more specifically if we would observe a difference between siFANCI-sensitive LNCaP and insensitive PC-3 cells, we analyzed mRNA levels of selected FA complex members in FANCI-silenced PC-3 cells using our RNA-sequencing data and RT-qPCR (**Supplementary Figures S4B, S5A**). We included the 22Rv1 cell line in the RT-qPCR analysis, as these cells also showed a reduced proliferation rate upon FANCI silencing like LNCaP cells but in cell cycle analysis behaved more like PC-3 cells, thus representing an interesting intermediate in response to FANCI depletion. Here, 22Rv1 cells showed the same trend as LNCaP cells with significant downregulation of FANCD2, FANCA/B/C/F, and UBE2T on the mRNA level (**Supplementary Figure S5B**). In PC-3 cells, FANCI silencing also appeared to reduce the mRNA levels of FANCD2/A/B/C/F but to a lesser extent (**Supplementary Figure S4B**). Interestingly, the baseline mRNA expression levels of the majority of FA pathway genes were lower in PC-3 cells compared to LNCaP cells based on RNA-sequencing data (**Supplementary Figures S4C, D**). In summary, these findings indicate that depletion of FANCI reduces the expression of FA core complex members in LNCaP cells, thus potentially making the cells more sensitive to DNA damage.

To analyze the effect of FANCI silencing on DNA damage and more specifically to see if LNCaP and 22Rv1 cells exhibit more DNA damage than PC-3 cells in response to FANCI silencing, we then stained siCTRL and siFANCI cells with antibodies against phosphorylated histone H2AX (γH2AX), known marker for DNA damage (46), in particular double-strand breaks (DSBs). Interestingly, in both siFANCI LNCaP and siFANCI 22Rv1 cells, no significant increase in gH2AX signal was detected in comparison to siRNA control, and the overall signal pattern was dispersed with very few weak gH2AX foci in both siFANCI and siCTRL cells, while in PC-3 cells, distinct punctate nuclear expression was observed especially in FANCI-silenced cells (**Figures 2C, D**, **Supplementary Figures 5C, S6**). This could be explained by the findings of another study where attenuation of γH2AX signal and disappearance of distinct γH2AX nuclear foci correlated with increased lethality, while when no reduction of γH2AX nuclear foci was observed, the cells handled DNA damage-inducing agents better (47). In our case, this could be interpreted such that in LNCaP and 22Rv1 cells the FANCI depletion results in reduced ability to respond to DNA damage as indicated by attenuated γH2AX signal, while PC-3 cells can recruit other FA-pathway independent DNA repair systems via γH2AX and thus continue proliferating normally.

### Activation of FANCI and FA pathway in response to carboplatin in prostate cancer cells

The key step in the activation of FA pathway-mediated DNA repair upon recognition of ICL-type DNA damage is the monoubiquitination of FANCI. As carboplatin is a known inducer of ICL-type DNA lesions, we analyzed its effect on FANCI monoubiquitination in prostate cancer cells using immunoblotting. Indeed, a band corresponding to the monoubiquitinated form of FANCI appeared in response to carboplatin in LNCaP cells (**Figure 2E**), indicating activation of the FA pathway. To observe if this applied to PC-3 cells, the ubiquitination of FANCI was also analyzed. Also, in PC-3 cells, FANCI appeared to be ubiquitinated in response to carboplatin (**Figure 2F**). Next, we wanted to find out if carboplatin exposure results in the formation of nuclear foci of FANCI since this has been shown to occur upon DNA damage and require the activation of FANCI through monoubiquitination and thus can be used as an indicator of FA pathway activation (2). Indeed, carboplatin exposure in LNCaP cells led to the formation of distinct punctate intranuclear FANCI protein-expression islands, while only sparse faint tiny foci are observed in PC-3 (**Figures 2G, H**). Furthermore, the overall expression of FANCI appeared to remain unaffected by carboplatin in PC-3 cells (**Figure 2H**). In LNCaP cells, in addition to the formation of the expected foci within the nucleus of the cells, the expression of FANCI is induced by carboplatin compared to dimethyl sulfoxide (DMSO) control (**Figure 2G**). These differences between the LNCaP and PC-3 cells indicate that while LNCaP cells appear to depend on FANCI and the FA pathway to protect themselves from the damaging effects of carboplatin, PC-3 cells potentially employ a different coping mechanism and are less dependent on FANCI and associated pathways. This is supported by the finding that no combinatorial effect of FANCI silencing and carboplatin was observed in PC-3 cells (**Supplementary Figure S7A**). Additionally, we observed some sensitivity to carboplatin in FANCI-silenced 22Rv1 cells (**Supplementary Figure S7B**), suggesting dependence on FANCI and the associated FA pathway that is potentially linked to their p53 WT/Q331R status.

### High expression of FA pathway genes correlates with poorer survival in prostate cancer patients

**Figure 3:**
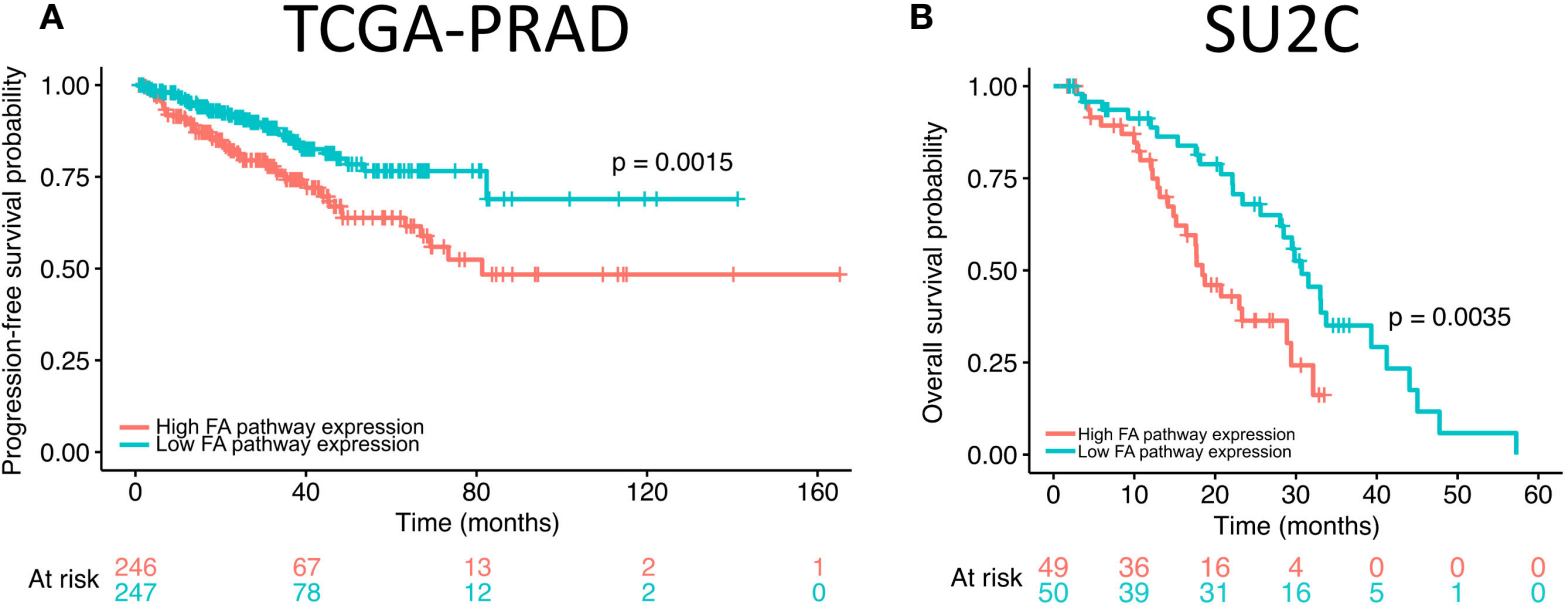
High expression of Fanconi anemia (FA) pathway genes correlates with progression-free and overall survival in prostate adenocarcinoma dataset. **(A)** The progression-free survival of high FA pathway gene expression (orange) in comparison to low (green) expression in The Cancer Genome Atlas (TCGA) prostate adenocarcinoma dataset. **(B)** Overall survival of high FA pathway gene expression (orange) in comparison to low (green) expression in Stand Up to Cancer (SU2C) metastatic castration-resistant prostate cancer dataset.

Our results revealed that FANCI silencing alters the expression of FA pathway genes in prostate cancer cells. To explore the potential role of FA pathway gene expression in prostate cancer patient outcome, we analyzed the overall and progression-free survival rates of patients with high and low expression of FA pathway genes using The Cancer Genome Atlas (TCGA) prostate adenocarcinoma (TCGA-PRAD; N = 498; (48)) and Stand Up to Cancer (SU2C; N = 444; (49)) metastatic castration-resistant prostate cancer cohorts. The Kaplan–Meier curves were plotted, and the results revealed that high expression of FA pathway genes significantly reduced progression-free survival in TCGA-PRAD dataset (**Figure 3A**) and overall survival in the SU2C dataset (**Figure 3B**). Taken together with the *in vitro* data, these findings suggest that high FA pathway expression is a targetable factor in a subset of prostate cancers.

### Genomic alterations of FA pathway members are prevalent in prostate adenocarcinoma patients

**Figure 4:**
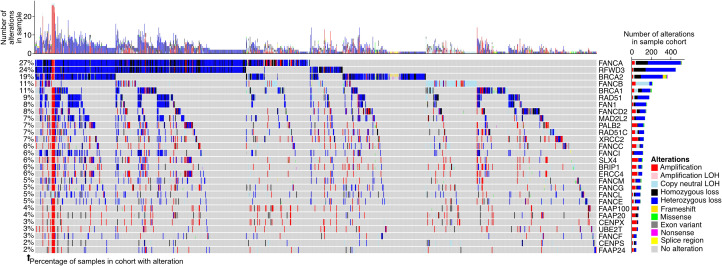
Fanconi anemia (FA) pathway gene alterations in prostate adenocarcinoma. Oncoprint displaying the predicted protein-altering mutations and copy number alterations in genes of the FA pathway in patients from The Cancer Genome Atlas (TCGA) prostate adenocarcinoma (TCGA-PRAD), PRAD-UK, PRAD-CA, PRAD-FR, PRAD-CN, Abida et al., and Grasso et al. cohorts (see Materials and Methods). Each column of the oncoprint is a sample, and only samples with at least one observed FA pathway gene alteration are shown. The bar plots show the number of alterations in each sample (top of oncoprint) and the number of alterations in a particular FA pathway gene across all samples (right of oncoprint). The percentage of samples with an alteration in each gene is indicated to the left of the oncoprint, calculated based on all samples in the seven included cohorts. Varying colors within the oncoprint and bar plots reflect the alteration types. LOH, loss of heterozygosity.

To further understand the degree to which the FA pathway is altered in prostate adenocarcinoma patients, we extracted the publicly available mutation and copy number information for the pathway genes in seven patient cohorts (48, 50–52) (**Figure 4**, N = 1,732 total tumor samples). We found 1,025 (59.2%) samples to have an alteration in at least one of the FA pathway genes, suggesting that genomic alteration of the pathway is a prominent feature in patients with the disease. FANCI was altered in 6.5% of samples, with the most prevalent alteration type being a heterozygous loss (78.8% of cases with FANCI alterations).

## Discussion

FANCI is a critical contributor to Fanconi anemia complex function in DNA damage response, and we have previously implicated FANCI as a potentially important mediator of treatment resistance in prostate cancer. We reported that FANCI-depleted LNCaP cells were more sensitive to carboplatin chemotherapy, a known inducer of ICL-type DNA damage, but the observed sensitivity was not studied any further. Our current study aimed to shed light on the functional role of FANCI in prostate cancer. To this end, we hypothesized that prostate cancer cells exploit FA pathway-mediated DNA repair to survive the DNA-damaging effects of chemotherapy and that FANCI plays a central role in this through its ability to regulate the accumulation of other FA core complex members to the damage site. FANCI is known to be important for the repair of ICL-type DNA damage induced by chemotherapy reagents and has been also linked to treatment resistance in some other cancers, but whether it plays a role in prostate cancer was previously unknown.

The FA pathway is particularly important for the replication-related repair of ICL-type DNA damage occurring during the S phase of the cell cycle. The initiation of FA-dependent ICL repair has been shown to involve the formation of the FANCM/FAAP24 subcomplex, which recognizes ICL lesions enabling the recruitment of other FA core complex components including FANCA/B/C/E/F/G and UBE2T (FANCT) (53, 54). FANCI and the associated FA pathway are important parts of DNA damage response, which is triggered by DNA lesions and normally involves the transcription factor p53 as an initiator of p21-dependent cell cycle arrest or apoptosis (30, 55). Interestingly, the regulation of the FA pathway and FANCI itself has been directly linked to both p53 and its target CDK inhibitor p21 (also known as p21WAF1/Cip1), which in turn is a negative regulator of the cell cycle mediating, among others, p53-dependent G1 growth arrest (56, 57). The activity of p53-p21 systems ensures that only cells with intact DNA will continue to the next phase in the cell cycle. Our results demonstrate that LNCaP but not PC-3 cells are sensitive to FANCI depletion-induced cell cycle arrest on G1, indicating that LNCaP cells are dependent on functional FANCI and the FA pathway for cell cycle progression. This could be linked to the p53 status of the cells, as LNCaP cells have functional wild-type p53, while FANCI depletion-insensitive PC-3 cells do not express p53 due to truncating mutations in *TP53* gene. It has, however, been recently suggested that both p53 and FANCI play roles in cell DNA repair/apoptosis switch decisions in response to DNA cross-links in colon cancer cells, although more mechanistic studies are needed to understand their interaction (55). Taken together, based on our results, we hypothesize that prostate cancer cells with functional p53 are dependent on FANCI and the FA pathway to maintain genomic stability during the cell cycle. However, more studies are required to elucidate the mechanism of how FANCI interacts with p53 and whether there is a negative/positive feedback loop that links FANCI activity to p53 activity in prostate cancer cells. Also, the molecular mechanisms behind FANCI’s role in cell cycle regulation need further research. Here, we also showed that FANCI affects the expression of FA pathway genes in prostate cancer cells. The FANCI-dependent prostate cancer cells were also more sensitive to carboplatin chemotherapy when FANCI was depleted, and this sensitivity might be linked to the p53 status of the cells. In addition, we were able to detect the carboplatin-induced FANCI monoubiquitination, indicating that FANCI becomes activated in response to chemotherapy in prostate cancer cells. Our results suggest that the activity of FANCI is needed for a subset of prostate cancer cells to survive under DNA damage-inducing chemotherapy, and FANCI depletion leads to the downregulation of multiple FA pathway genes and thus most likely to the inactivation of the FA pathway in prostate cancer.

Interestingly, the joint amount of FA gene mutations and FA copy number variations is as high as 50% (mainly amplification) in patients with TCGA genome data (58, 59). In the current study, we analyzed the different FA pathway gene alterations in detail and concluded that 59.2% of prostate cancer patient tumors displayed mutations or alterations in one or more of the FA pathway genes, suggesting that genomic alteration of the pathway is a prominent, previously unheralded feature in patients with the disease. Our data suggest that FANCI function is a critical contributor to resistance to DNA-damaging chemotherapy in a potentially clinically identifiable subset of prostate cancers.

Specifically, these findings support further development of pharmacologic inhibitors of FANCI and the pursuit of clinical trials comparing outcomes in patients with and without FANCI inhibition together with carboplatin or other DNA-damaging chemotherapy in patients with metastatic prostate cancers harboring wild-type p53, which is approximately 40% of castration-resistant metastatic prostate cancers (mCRPC) according to a recent study (60).

Our results also suggest that FANCI could play a major role in regulating overall FA pathway response and could be a potential drug target in prostate cancer cells. We propose further studies to understand the mechanisms of how FANCI as a DNA binding protein, which together with FANCD2, recognizes ICL breaks can also modulate the expression of the whole complex of FA pathway genes required for the FA pathway activity. One intriguing possibility is that FANCI as a DNA-binding protein could act as a transcription factor as already suggested by Sondalle et al. (61). Their study shows that FANCI has an alternative function outside of DNA repair in ribosome biogenesis, specifically in the transcription of pre-ribosomal RNA (pre-rRNA) and in large ribosomal subunit (LSU) pre-rRNA processing, and as, one potential mechanism, suggest the ability of FANCI to act as a transcription factor for RNA polymerase I. The potential role of FANCI as a transcriptional regulator as part of the FA complex is further supported by a study from Tremblay et al. demonstrating that the Fanconi anemia core complex can function as a transcriptional co-regulator via modulation of Hairy Enhancer of Split 1 (HES1) gene transcription (62). As HES1 is a transcriptional repressor, FA complex can thus alter the expression of HES1 target genes, including p21^cip1/waf1^.

In conclusion, here we identify FANCI as a potential regulator of FA pathway member expression and activity in prostate cancer and suggest that targeted therapies against FANCI could be designed and tested for the prevention of FA pathway response in treatment resistance in prostate and potentially also in other cancers.

## Materials and methods

### Cell culture

LNCaP, 22Rv1, and PC-3 cells were cultured in a humidified CO_2_ incubator at 37°C in Gibco™ RPMI 1640 (1×) media (Cat. no. 31870-025, Life Technologies Limited, Carlsbad, CA, USA) supplemented with 10% fetal bovine serum (FBS; Cat. no. 10270-106, Thermo Fisher Scientific, Waltham, MA, USA), 2 mM of l-glutamine (Cat. no. 25030-024, Gibco®, Thermo Fisher Scientific), and combination of 100 U/ml of penicillin and 100 µg/ml of streptomycin (Cat. no. 10378-015, Gibco® Pen Strep, Thermo Fisher Scientific). PC-3, LNCaP, and 22Rv1 cell lines were obtained from ATCC (Manassas, VA, USA) in 2011. LNCaP and 22Rv1 cell lines were authenticated using Promega short tandem repeat (STR) systems referenced to the ATCC STR database by the Johns Hopkins Genetic Resources Core Facility (JHGRCF). PC-3 cell line was authenticated at the Genomics Unit of Technology Centre in the Institute for Molecular Medicine Finland (FIMM) using the Promega GenePrint24 System.

For proliferation assays conducted on a 384-well plate, 22Rv1 and PC-3 cells were seeded at an initial density of 1,500 cells per well. The addition of carboplatin (Cat. no. S1215, Selleck Chemicals GmbH, Munich, Germany) on the cells was performed 24 hours after siRNA addition. Prior to the addition, the drugs were diluted from DMSO-based stock solutions to 6× solutions in an LNCaP culture medium without FBS. DMSO (Cat. no. D8418-250ML, Merck, Darmstadt, Germany) was added to the control cells to a final concentration corresponding to the highest DMSO concentration (0.2%) in the carboplatin-exposed cells. Cell growth monitoring and analysis of the cell confluency were performed using the IncuCyte S3 Live-cell Analysis System (Sartorius, Goettingen, Germany).

The culturing of all cell lines cells was performed on six-well plates for the collection of RNA and protein samples for reverse transcription quantitative real-time PCR (RT-qPCR) and Western blotting analysis, respectively. The seeding density was 3.5 × 10^5^ cells per well. The addition of the drugs was performed after a 24-hour initial incubation period, and for the preparation of the drug dilutions, a culture medium without FBS was used. To control cells, DMSO diluted in drug dilution media was added to a final concentration of 0.02%.

To obtain cells for flow cytometry analysis, the culturing was performed using a six-well plate format and a seeding density of 3.0 × 10^5^ cells per well. The cells were cultured for 96 hours prior to preparation for flow cytometry analysis.

### Cell cycle cell lines for live cell imaging

The LNCaP, PC-3, and 22Rv1 cells were initially plated on a 48-well at a seeding density of 10,000 cells/well for lentiviral transductions with IncuCyte Cell Cycle Green/Red Lentivirus Reagent (Cat. no. 4779, Sartorius). The cells were transduced using a multiplicity of infection (MOI) of 2.5, and after 1 week of culturing, puromycin at a concentration of 1 µg/ml (LNCaP and 22Rv1) or 2 µg/ml (PC-3) was used to select only successfully transduced cells. For the live cell imaging analysis on a 96-well plate, a seeding density of 5,000 cells/well was used, and cells were siRNA transfected using the reverse transfection method. Cells were imaged, and the amount of red and green fluorescent cells was quantified using IncuCyte S3 and the associated analysis software.

### siRNA transfections

Silencing RNA experiments used a reverse transfection protocol. Cells were transfected either with non-targeting siRNA (Cat. no. D-001810-10-20, siCTRL, ON-TARGETplus Non-targeting Control Pool, Dharmacon, Lafayette, CO, USA) or FANCI silencing RNA (Cat. no. L-022320-01-0005, human FANCI ON-TARGETplus SMARTpool siRNA, Dharmacon) to 25 nM of final siRNA concentration using Opti-MEM™ I (Cat. no. 31985-047, Thermo Fisher Scientific) and Lipofectamine RNAiMAX (Cat. no 13778150, Invitrogen, Carlsbad, CA, USA).

### Flow cytometry analysis

On day 4 after siRNA transfection, the cells were detached using trypsin, washed with 1× phosphate-buffered saline (PBS), and fixed overnight at +4°C using 70% ethanol. After ethanol removal, the cells were resuspended in 1× PBS stained with propidium iodide in the presence of RNase for 1 hour at +37°C. The cells were analyzed in 1× PBS with 2% FBS included. The fluorescence of cells was measured using NovoCyte Quanteon Flow Cytometer (Agilent, Santa Clara, CA, USA) with NovoExpress® software (Agilent). The analysis of the results was performed using NovoExpress software.

### Reverse transcription quantitative real-time PCR (RT-qPCR)

The isolation of RNA was conducted using TriPure Isolation Reagent (Cat. no. 11667165001, Roche, Basel, Switzerland) following the manufacturer’s protocol. The concentration of the RNA samples was measured using NanoDrop™ One/OneC Microvolume UV-Vis Spectrophotometer (Thermo Fisher Scientific), followed by dilution of the samples to 1 µg/µl. The conversion of 1 µg of RNA to cDNA was performed using Transcriptor First Strand cDNA Synthesis Kit (Cat. no. 4897030001, Roche) according to the manufacturer’s instructions.

The RT-qPCR run was performed using LightCycler™ 480 SYBR Green I Master (Cat. no. 04887352001, Roche) and The LightCycler® 480 Real-Time PCR System (Roche) with 96-multiwell format. Primer pairs targeting FANCI, FANCD2, FANCA, FANCB, FANCC, FANCF, and UBE2T were used (**Supplementary Table S1**). Each RT-qPCR run included three biological and two technical replicates per treatment with fold change calculated based on the obtained Ct-values. Normalization was performed against the GAPDH values measured.

### RNA-sequencing and pathway analyses

Purified RNA samples from the FANCI siRNA (n = 3) and non-targeting siRNA (n = 3) exposed LNCaP and PC-3 cells were sent to Novogene (Cambridge, UK) for quality check, library preparation, and sequencing. Sequenced raw reads were aligned to the hg38 genome using STAR2.7 (63) using default settings with max 10 mismatches and max 10 multi-mapped reads. Differentially expressed genes were analyzed with DESeq2 (64) through HOMER (65). The total count per gene was calculated using transcripts per million (TPM) normalization. Differentially expressed gene sets were subjected to GSEA using GSEA software (v.4.2.2) (The Broad Institute, Massachusetts Institute of Technology, Cambridge, MA, USA) (27, 28). Normalized enrichment score (NES) <−2, false discovery rate (FDR) <0.25, and p-value <0.05 were used as cutoffs. The generated RNA-seq datasets were submitted to the NCBI Gene Expression Omnibus database (http://www.ncbi.nlm.nih.gov/geo/), accession code: GSE211363.

### Western blotting

Whole-cell lysates were prepared using sodium dodecyl sulfate (SDS) sample buffer (66 mM of Tris-HCl, pH 6.8, 13% glycerol, 2.1% SDS, and 0.01% bromophenol blue) with protease inhibitor added (Cat. no. 11697498001, cOmplete™ Protease Inhibitor Cocktail, Roche). Proteins were separated by SDS–polyacrylamide gel electrophoresis (SDS-PAGE) and wet transferred in methanol-based transfer buffer (20% v/v methanol, 25 mM of Tris, 192 mM of glycine) onto nitrocellulose membrane (Cat. no. 88018, Thermo Fisher Scientific). Blocking was conducted at room temperature for 1 hour using 5% non-fat dry milk in 1× TBS-Tween (20 mM of Tris-Cl, 137 mM of NaCl, and 0.1% Tween 20) after which primary antibodies were applied overnight at 4°C. Primary antibody solutions were prepared in a blocking buffer. For anti-FANCI (Cat. no. PA5-59014, Invitrogen) and for anti-FANCD2 (Cat. no. sc-20022, Santa Cruz Biotechnology, Dallas, TX, USA), dilution ratio 1:500 was used; for anti-FANCB (Cat. no. 14243, Cell Signaling, Danvers, MA, USA), anti-UBE2T (Cat. no. 10105-2-AP Thermo Fisher Scientific/ProteinTech, Chicago, IL, USA), anti-p53 (Cat. no. ab1101, Anti-p53 antibody [DO-1] – ChIP Grade, Abcam, Cambridge, UK), and anti-p21 (Cat. no. 10355-1-AP, P21 Polyclonal antibody, ProteinTech), dilution ratio 1:1,000 was used; for anti-GAPDH (Cat. no. sc-25778, Santa Cruz Biotechnology), dilution ratio 1:5,000 was used. Secondary antibody dilutions were prepared using 1× TBS-Tween. Goat anti-rabbit IgG horseradish peroxidase conjugate (Cat. no. G21234, Invitrogen) in dilution ratio of 1:10,000 and goat anti-mouse IgG horseradish peroxidase conjugate (Cat. no. G21234, Invitrogen) in dilution ratio 1:5,000 were used as the secondary antibody. Pierce™ ECL Western Blotting Substrate (Cat. no. 32106, Thermo Fisher Scientific) was used for detection, and imaging was performed with the Chemidoc Imaging system (Bio-Rad, Hercules, CA, USA).

### Immunofluorescence imaging

LNCaP and PC-3 cells exposed to either DMSO or carboplatin (final concentration 5 µM) or either FANCI or non-targeting control siRNA were fixed using 4% paraformaldehyde, followed by permeabilization with 0.1% Triton-X and overnight incubation at 4°C with primary antibody. For FANCI antibody (Cat. no. PA5-59014, Human FANCI Polyclonal Antibody, Invitrogen) and phosphorylated Histone 2AX (γH2AX, Cat. no. 5438, Phospho-Histone H2A.X, rabbit, Cell Signaling), 1:100 dilution was used. A secondary antibody was applied simultaneously with nuclear staining DAPI (Cat. no. D8417-1MG, Sigma, St. Louis, MO, USA). The secondary antibody was applied at room temperature (RT) using a 1:500 dilution ratio. Alexa Fluor 633 anti-rabbit antibody (Alexa Fluor™ 633 Goat anti-Rabbit IgG (H+L), cat. no. A21070) was used as the secondary antibody. Imaging was performed with a Zeiss LSM 800 confocal microscope at ×63 magnification.

### Analysis of FA pathway alterations in prostate adenocarcinoma patient cohorts

Gene expression data and patient outcome information were downloaded from cBioPortal v4.1.9 for TCGA-PRAD and SU2C metastatic castration-resistant prostate cancer datasets (48, 49). The GSVA package v1.34.0 in R v3.6.3 was used to score each patient for the expression of the set of FA pathway genes. Patients were stratified into low and high FA pathway score groups based on the median GSVA score. Survival analyses were performed using the survival package v3.2-3, and Kaplan–Meier curves were plotted using the survminer package v0.4.8. The two-sided log-rank test was used to assess for significance in survival analyses.

Publicly available somatic mutation and copy number data were downloaded for seven prostate adenocarcinoma cohorts from cBioPortal v4.1.9 (TCGA-PRAD, Abida et al., Grasso et al.) and the ICGC Data Portal release 28 (PRAD-UK, PRAD-CA, PRAD-FR, and PRAD-CN) (48, 50–52). Samples with more than 10 mutations per megabase were excluded as likely hypermutators. For patients with multiple samples (e.g., from multiple metastases), one of the samples was chosen randomly to represent the patient. For each cohort, somatic mutations were restricted to those predicted to be protein-altering. These were missense, exon, non-sense, frameshift, and splice region variants. Copy number segment coordinates reported for the ICGC cohorts were intersected with the hg19 coordinates of the Fanconi anemia pathway genes to determine the copy number status of each gene using the TxDb.Hsapiens.UCSC.hg19.knownGene package v3.2.2 and the GenomicRanges package v1.38.0 in R v3.6.3. Copy number data were not available for the PRAD-CN cohort, and no copy number alteration type was reported for the PRAD-FR cohort; thus, copy number information from these cohorts was not included in the analyses and visualizations. Copy number calls from cBioPortal distinguished between amplifications and low-level copy number gains, and for these cases, the low-level gains were not included in analyses or visualizations. Oncoprints of alterations in each cohort and the overall sample set were constructed using the ComplexHeatmap R package v2.2.0.

## Data availability statement

The datasets presented in this study can be found in online repositories. The names of the repository/repositories and accession number(s) can be found in the article method section.

## Author contributions

HK: Conceptualization, Data curation, Formal Analysis, Funding acquisition, Investigation, Methodology, Validation, Visualization, Writing – original draft, Writing – review & editing. ST: Data curation, Formal Analysis, Investigation, Methodology, Validation, Visualization, Writing – review & editing. RK: Data curation, Methodology, Validation, Writing – review & editing. ET: Data curation, Formal Analysis, Methodology, Validation, Writing – review & editing. VP: Data curation, Validation, Writing – review & editing. MN: Conceptualization, Data curation, Formal Analysis, Investigation, Writing – review & editing. GB: Conceptualization, Formal Analysis, Investigation, Writing – review & editing. KK: Conceptualization, Data curation, Formal Analysis, Funding acquisition, Investigation, Methodology, Project administration, Resources, Supervision, Validation, Visualization, Writing – original draft, Writing – review & editing.
