# Supplementary material for: Fanconi anemia pathway regulation by FANCI in prostate cancer
Source: Front Oncol. 2023 Oct 30;13:1260826. doi: 10.3389/fonc.2023.1260826 (PMC10643534; doi:10.3389/fonc.2023.1260826)
Supplement: Supplementary file 1 [file DataSheet_1.pdf]

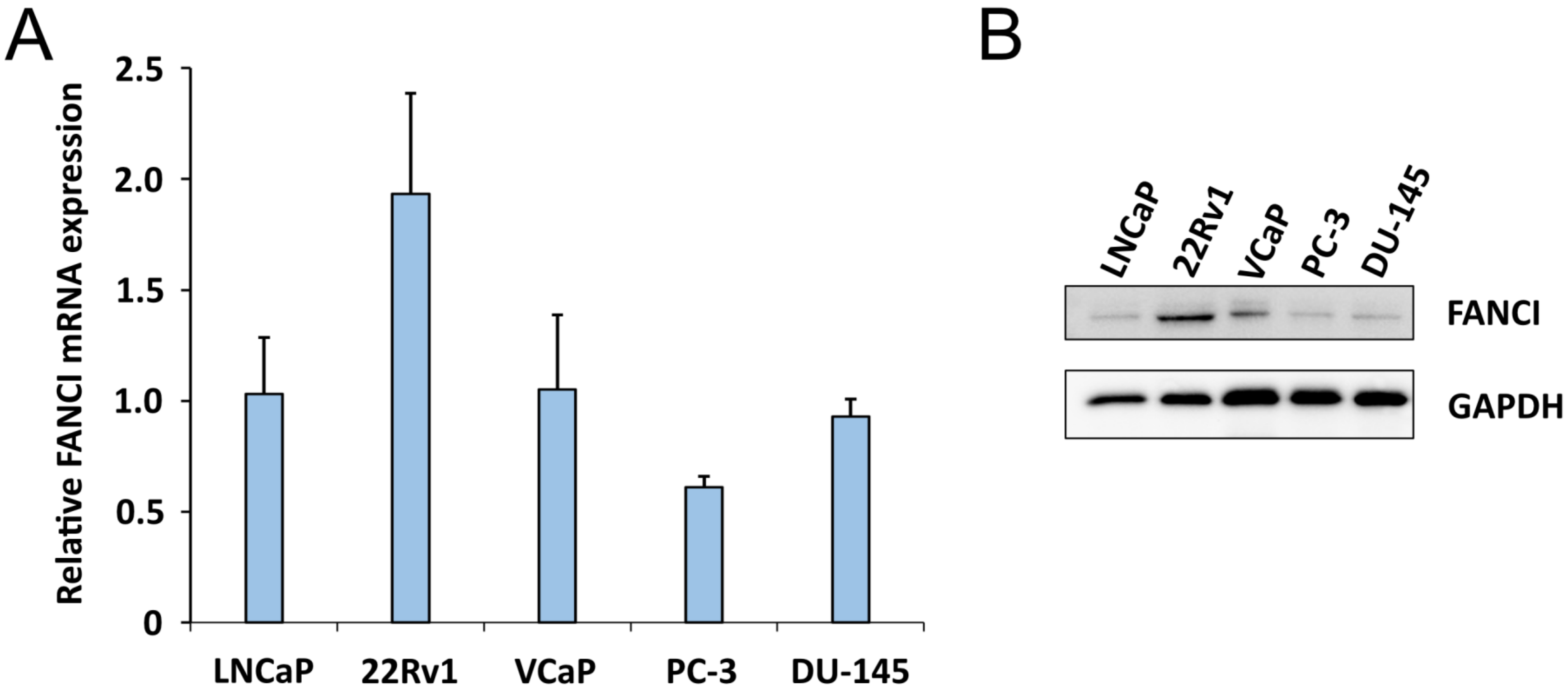

Supplementary figure S1. FANCI expression in prostate cancer cell lines. A) A bar graph indicating the FANCI mRNA expression levels in prostate cancer cell lines LNCaP, 22Rv1, VCaP, PC-3 and DU-145 based on qPCR analysis. B) FANCI expression in prostate cancer cell lines analysed using western blot. Images of the whole membranes are found in Supplementary data.

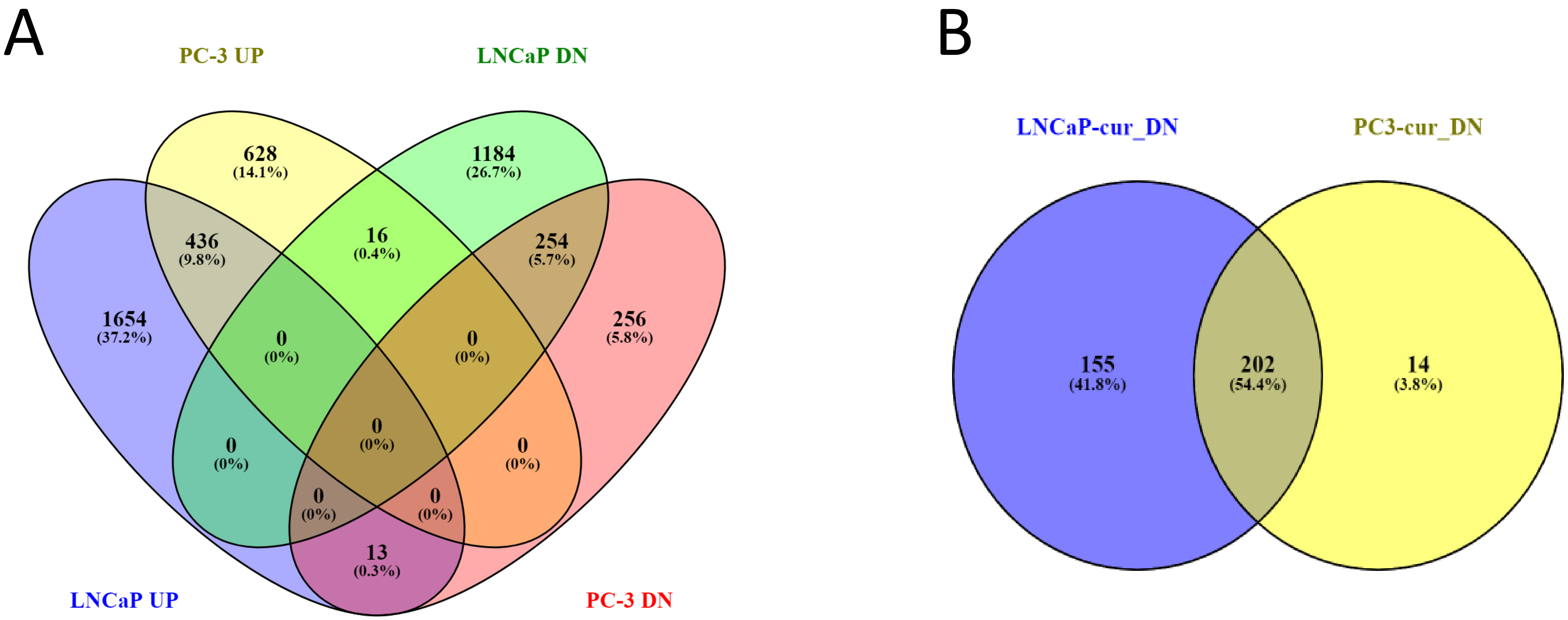

Supplementary figure S2. Significantly greater impact on pathway up- and downregulation by FANCI silencing in LNCaP as compared to PC-3 prostate cancer cells. A) The number of both up- and downregulated genes is significantly larger in LNCaP than in PC-3 cells in response to FANCI silencing when log fold cut off 0.5/-0.5 was used (adj. P-value <0.05). B) Based on the GSEA analysis on the curated gene sets, there are significantly larger number of gene sets exclusively downregulated in LNCaP cells (155 sets) in comparison to those downregulated exclusively in PC-3 cells (14 sets) when FANCI is silenced. Comparison and diagrams done using Venny tool (Oliveros, J.C. (2007-2015) Venny. An interactive tool for comparing lists with Venn's diagrams. https://bioinfogp.cnb.csic.es/tools/venny/index.html).

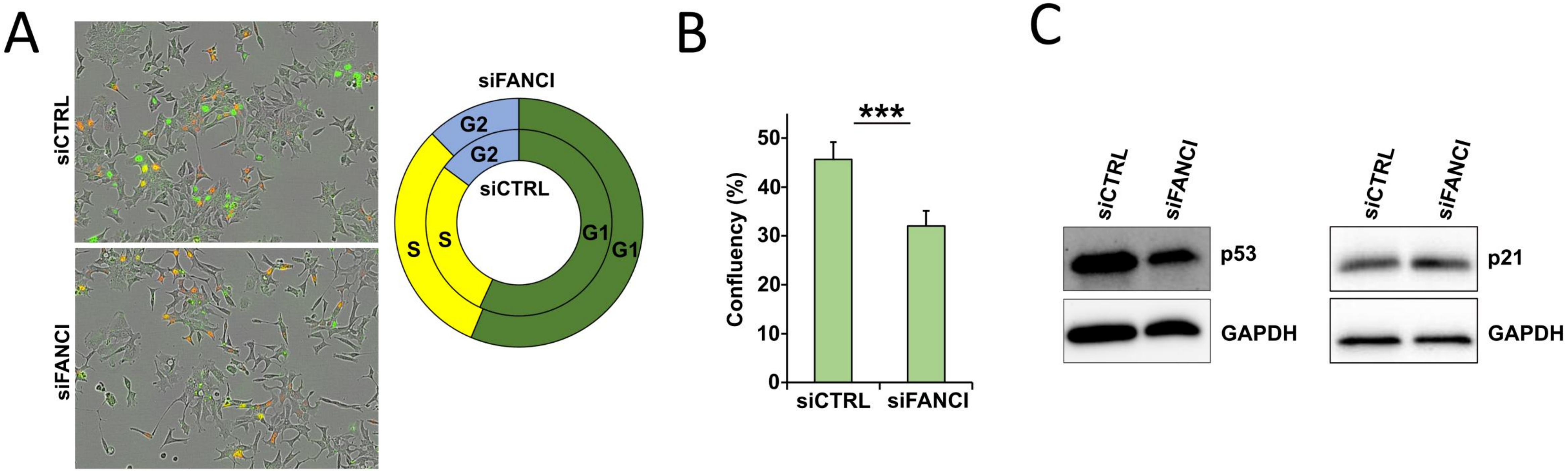

Supplementary figure S3. FANCI silencing has a proliferative effect on p53-expressing 22Rv1 cells. A) No difference between FANCI silenced 22Rv1 cells and control cells (siCTRL) are observed in Incucyte live cell imaging using 22Rv1 cells with fluorescent labeled cell cycle markers Cdt1 (red, G1 phase cells) and geminin (green, G2 phase cells). The S phase cells have both Cdt1 and geminin and thus appear yellow. On the left: Incucyte images of the control siRNA and FANCI silenced 22Rv1 cells. On the right: Donut plot of the percentages of cells in G1 (green section), S (yellow section) and G2 (blue section) phases based on analysis with Incucyte software (Inner circle: siCTRL, Outer circle: siFANCI). B) FANCI depletion, however, does reduce the proliferation rate of 22Rv1 cells based on cell confluency analysis using Incucyte analysis software. Bars represent mean±SD with n=6. p-values shown as asterisks (*p≤ 0.05, **p ≤ 0.01 and ***p ≤ 0.001). C) Western blot analysis of p53 and p21, known cell cycle regulators, in FANCI silenced or control siRNA 22Rv1 cells showing downregulation of p53 in 22Rv1 cells in response to FANCI silencing while no marked difference in p21 expression is observed between control (siSCTRL) and FANCI silenced (siFANCI) cells. GAPDH was used as reference. White space was used to make explicit for the grouping of blots cropped from different gels. Full scan of blots are found in Supplementary data.

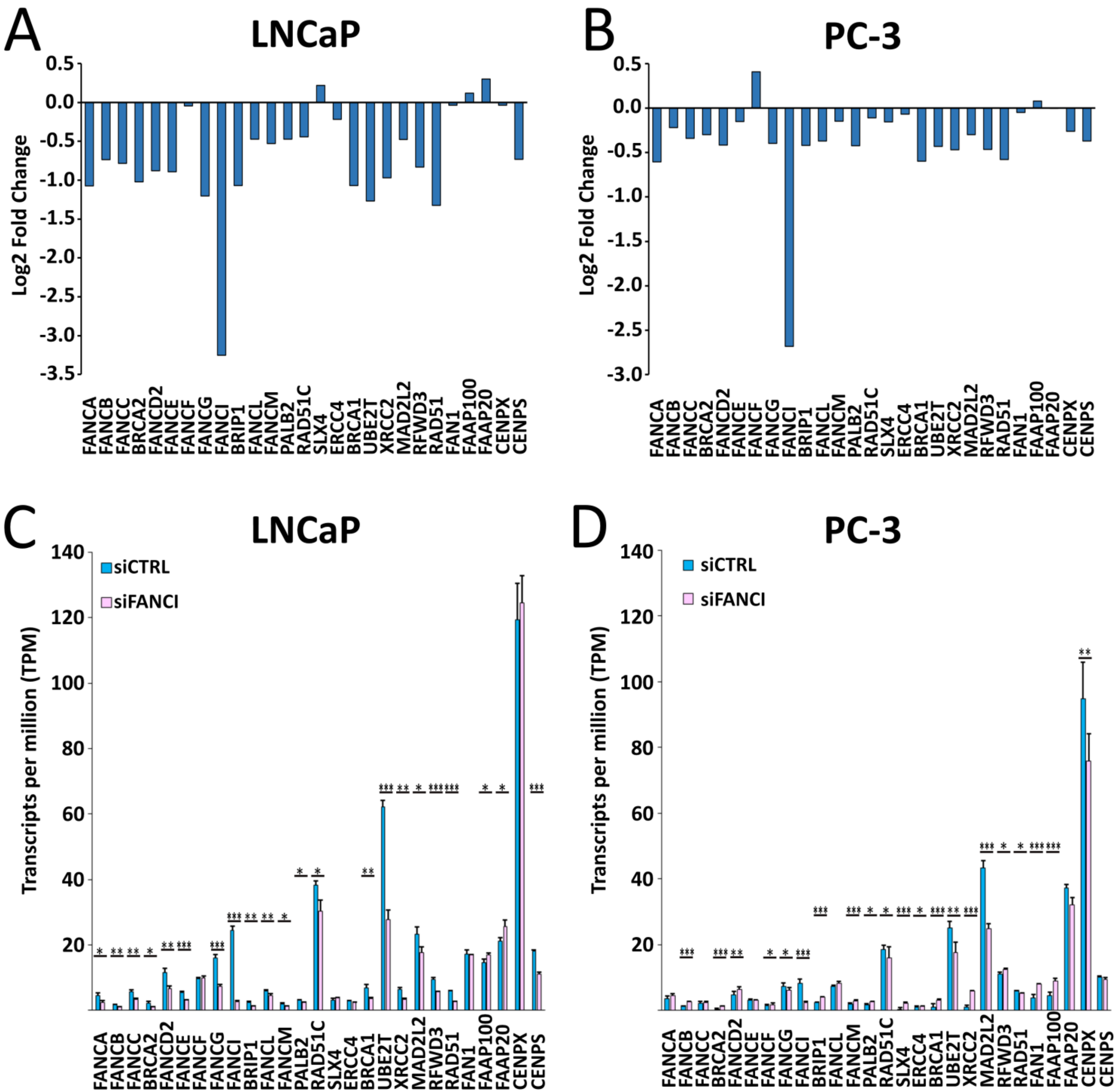

Supplementary figure S4. FA pathway gene expression in LNCaP and PC-3 cells with siFANCI based on RNA-sequencing. Fanconi anemia complex member mRNA levels upon FANCI silencing. A) FA pathway genes are mostly downregulated in FANCI silenced LNCaP cells based on log fold change from RNA-sequencing. B) In PC-3 cells the downregulation of FA pathway genes is more modest when compared to LNCaP. C) The number of transcripts for FA pathway members in LNCaP cells (siCTRL, blue bars; siFANCI, pink bars). Bars represent mean±SD with n=3. p-values shown as asterisks (*p≤ 0.05, **p ≤ 0.01 and ***p ≤ 0.001). D) The number of transcripts for FA pathway members in PC-3 cells (siCTRL, blue bars; siFANCI, pink bars). Bars represent mean±SD with n=3. p-values shown as asterisks (*p≤ 0.05, **p ≤ 0.01 and ***p ≤ 0.001).

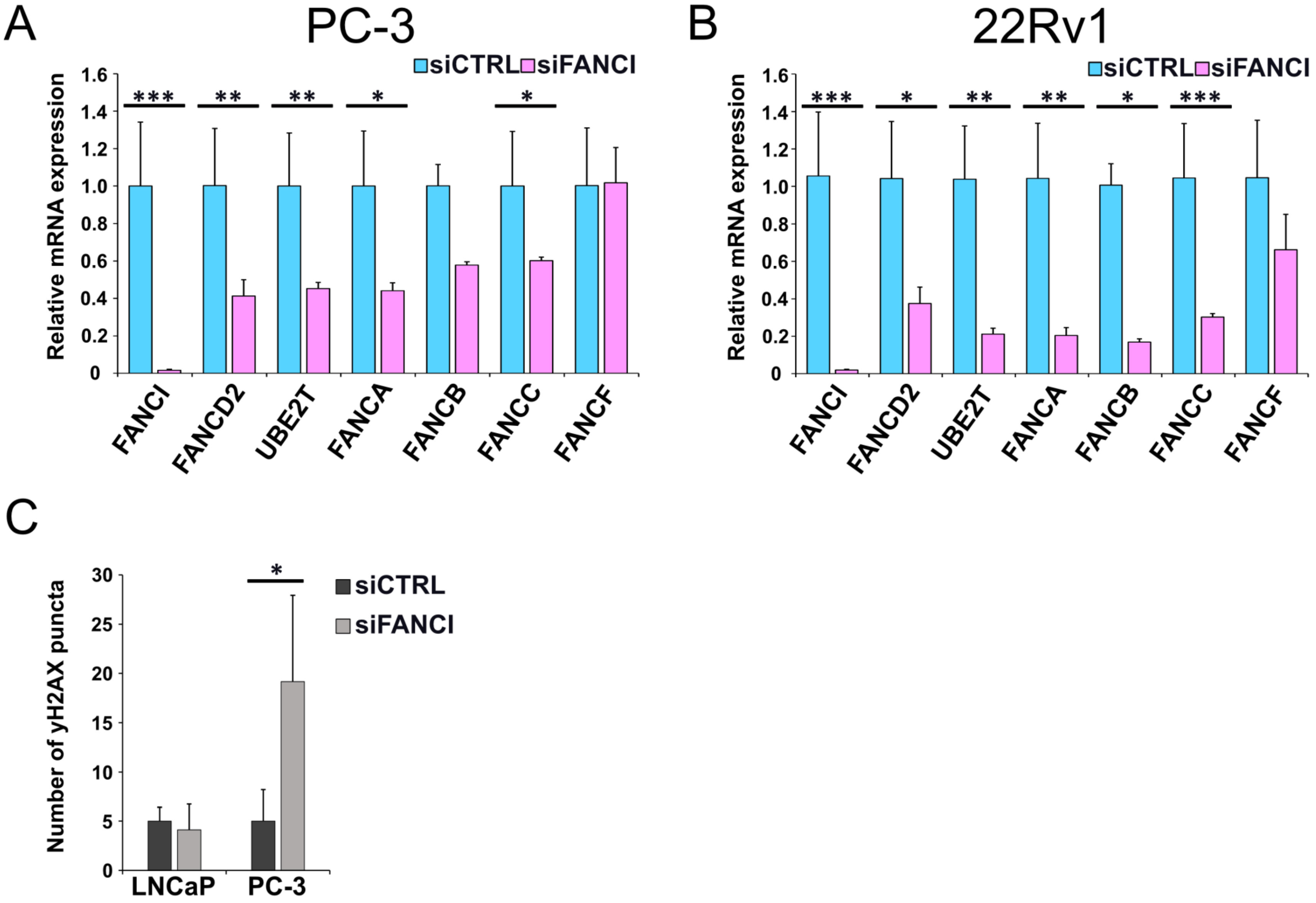

Supplementary figure S5. FANCI RNA silencing drives down the expression of other FA complex members in prostate cancer cell lines and sensitizes 22Rv1 but not PC-3 cells to Carboplatin. A) The mRNA expression levels of FA core complex members FANCI/D2/A/B/C/F and UBE2T were analyzed using qPCR from FANCI silenced 22Rv1 cells (siFANCI, pink bars). Cells exposed to non-targeting control siRNA (siCTRL; blue bars) were used as control. FANCI silencing downregulated the mRNA levels of the FA core complex members. B) In PC-3 cells the effect was more moderate than in the other cell lines included in the analysis. Bars represent mean±SD with n=3. p-values shown as asterisks (*p≤ 0.05, **p ≤ 0.01 and ***p ≤ 0.001).
C) Quantification of γH2AX puncta from LNCaP and PC-3 cells with control (siCTRL) or FANCI siRNA (siFANCI). Bars represent mean±SD with n=31. p-values shown as asterisks (*p≤ 0.05, **p ≤ 0.01 and ***p ≤ 0.001).

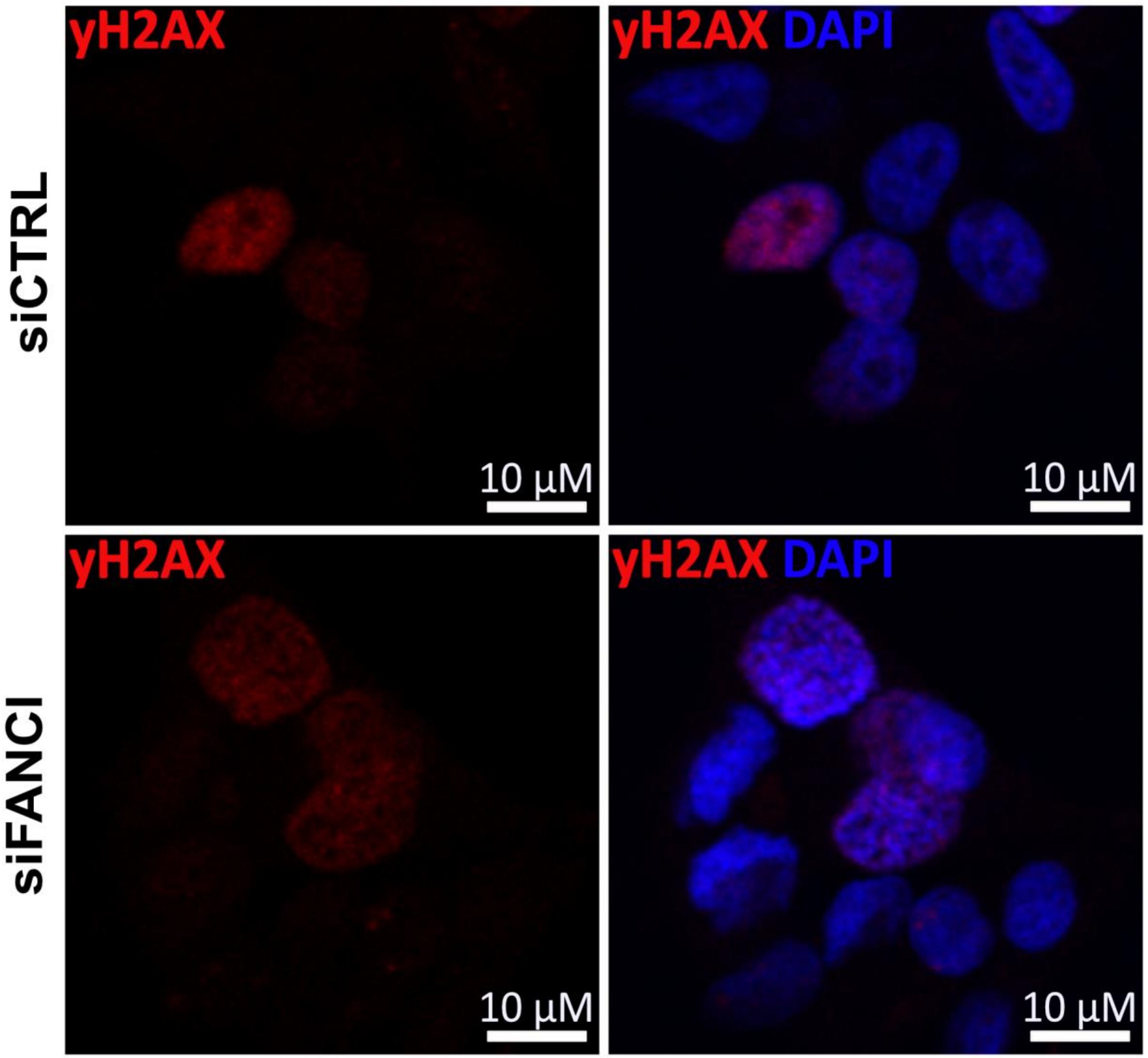

Supplementary figure S6. FANCI depletion attenuates the expression of DNA damage marker phosphorylated Histone 2AX (yH2AX). Immunofluorescence images of 22Rv1 cells with either FANCI or control siRNA stained with yH2AX (shown in red), which is a known marker for DNA damage. DAPI staining (shown in blue) was used to visualize the nuclei of the cells. Attenuation of yH2AX could be an indication of reduced ability to activate DNA damage response, which yH2AX is also part of.

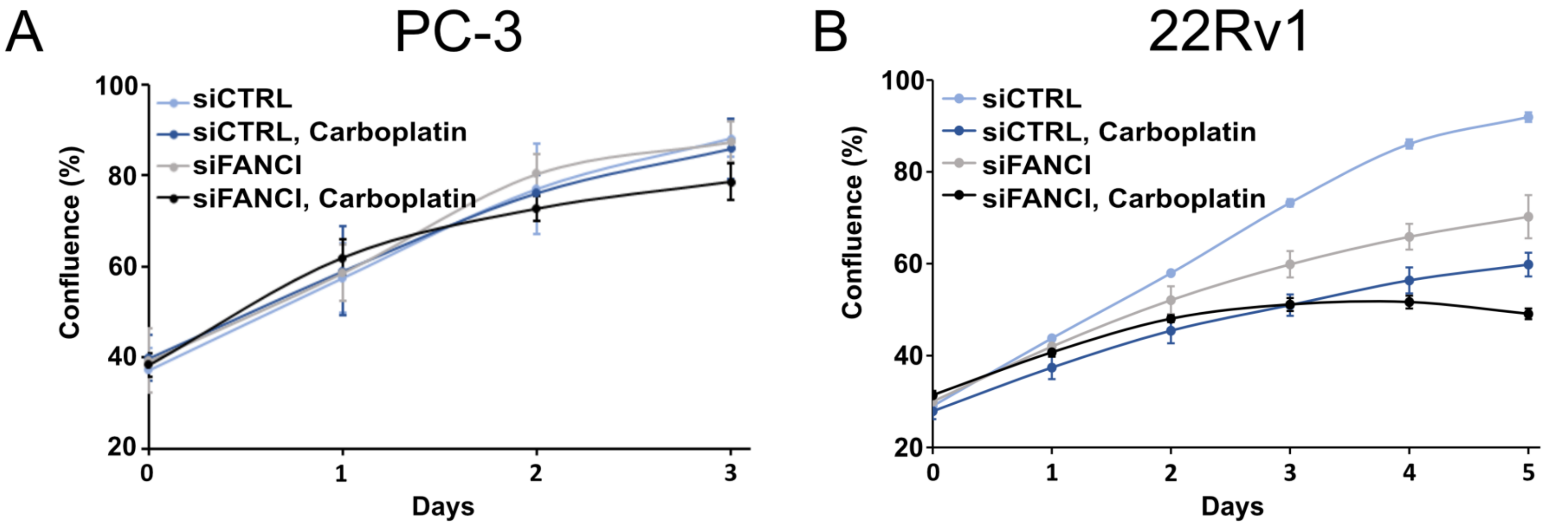

Supplementary figure S7. FANCI RNA silencing drives down the expression of other FA complex members in prostate cancer cell lines and sensitizes 22Rv1 but not PC-3 cells to Carboplatin. A) PC-3 proliferation in cells exposed to Carboplatin at concentration 5 µM was analysed in the presence of either control non-targeting siRNA (siCTRL; light blue curve for siCTRL alone and dark blue for siCTRL in combination with Carboplatin) or FANCI silencing RNA (siFANCI; gray for siFANCI alone and black for siFANCI together with Carboplatin). B) Proliferation of 22Rv1 cells when exposed to Carboplatin (5 µM final concentration) in combination with siCTRL (light blue curve for siCTRL alone and dark blue for siCTRL in combination with Carboplatin) or siFANCI (gray for siFANCI alone and black for siFANCI together with Carboplatin). Analysis was done using Incucyte live cell imaging and analysis platform.
