# Supplementary material for: Fanconi anemia pathway regulation by FANCI in prostate cancer
Source: Front Oncol. 2023 Oct 30;13:1260826. doi: 10.3389/fonc.2023.1260826 (PMC10643534; doi:10.3389/fonc.2023.1260826)
Supplement: Supplementary file 2 [file DataSheet_2.pdf]

Supplementary Table S1. RT-PCR primer sequences, their T_m-values and source for the sequence.

Primer name	Sequence	T_m-values (°C)	Source
GAPDH Forward	CATGAGAAGTATGACAACAGCCT	54.8	(1)
GAPDH Reverse	AGTCCTTCCACGATACCAAAGT	55.8	
FANCI Forward	CCACCTTTGGTCTATCAGCTTC	55.4	(2)
FANCI Reverse	CAACATCCAATAGCTCGTCACC	55.8	
FANCD2 Forward	AGACTGTCAAAATCTGAGGATAAAGAGA	55.5	(3)
FANCD2 Reverse	TGGTTGCTTCCTGGTTTTGG	56.1	
FANCA Forward	GTTGCCTCTAGCGTGGGAC	58.2	(4)
FANCA Reverse	GGAGAACATACTGTGTGCCAAT	54.9	
FANCB Forward	CAACTTATGGATTCAGGTGGAG	53.1	(5)
FANCB Reverse	TTTCCCATTTAGCAGCAACC	53.6	
FANCC Forward	GGAAATCCTCCAGCCAGAGT	56.7	(6)
FANCC Reverse	ATCAACAACCCGGAATATGG	52.6	
FANCF Forward	TGCTAACAGACTGGGGTCAAC	57.0	(7)
FANCF Reverse	TACAGGTCTCCAGGGCAGTTA	57.3	

1. Bai Q, Li X, Wang X, Xu Y, Wang L, Zhang Q, et al. VEGF is involved in the increase of dermal microvascular permeability induced by tryptase. ISRN Dermatol. 2012;2012:941465.
2. Zheng P, Li L. FANCI Cooperates with IMPDH2 to Promote Lung Adenocarcinoma Tumor Growth via a MEK/ERK/MMPs Pathway. OncoTargets Ther. 2020 Jan;Volume 13:451–63.
3. Jaber S, Toufektchan E, Lejour V, Bardot B, Toledo F. p53 downregulates the Fanconi anaemia DNA repair pathway. Nat Commun. 2016 Apr 1;7:11091.
4. Oku Y, Nishiya N, Tazawa T, Kobayashi T, Umezawa N, Sugawara Y, et al. Augmentation of the therapeutic efficacy of WEE1 kinase inhibitor AZD1775 by inhibiting the YAP-E2F1-DNA damage response pathway axis. FEBS Open Bio. 2018 Jun;8(6):1001–12.
5. Sun D, Zong Y, Cheng J, Li Z, Xing L, Yu J. GINS2 attenuates the development of lung cancer by inhibiting the STAT signaling pathway. J Cancer. 2021;12(1):99–110.
6. Pouliot GP, Degar J, Hinze L, Kochupurakkal B, Vo CD, Burns MA, et al. Fanconi-BRCA pathway mutations in childhood T-cell acute lymphoblastic leukemia. Coppola V, editor. PLOS ONE. 2019 Nov 13;14(11):e0221288.
7. Dai CH, Li J, Chen P, Jiang HG, Wu M, Chen YC. RNA interferences targeting the Fanconi anemia/BRCA pathway upstream genes reverse cisplatin resistance in drug-resistant lung cancer cells. J Biomed Sci. 2015 Sep 18;22(1):77.
