# Supplementary material for: Fanconi anemia pathway regulation by FANCI in prostate cancer
Source: Front Oncol. 2023 Oct 30;13:1260826. doi: 10.3389/fonc.2023.1260826 (PMC10643534; doi:10.3389/fonc.2023.1260826)
Supplement: Supplementary file 3 [file DataSheet_3.pdf]

Supplementary table S2. Molecular Signature Database gene sets and the corresponding leading-edge subset of genes from LNCaP siFANCI GSEA analysis

Reactome gene set	NES-value	Genes contributing to the enrichment
FISCHER_G2_M_CELL_CYCLE	-4.2	NUDT7, MSH6, UNG, JUND, UBR7, MASTL, GMNN, USP1, RFC2, CCNE2, TMEM243, ACYP1, CDC25A, CENPQ, YEATS4, TIFA, ATAD2, FANCE, MSH5-SAPCD1, NASP, SLBP, MCM6, RFXAP, DCLRE1B, ASPH, CLSPN, CDT1, MCM5, EZH2, POLD3, CHAF1A, PCNA, POLE2, TIPIN, E2F2, DSCC1, CHAF1B, WDHD1, CDC7, E2F1, MCM3, FAM111B, CHEK1, RFC4, PRIM1, E2F7, RECQL4, BRIP1, E2F8, BRCA1, MCM2, GINS2, RRM2, TCF19, WDR76, RBBP8, CDCA7, FANCG, MCM10, ZNF367, CDC6, HELLS, EXO1, RAD51, DTL, FEN1, POLA2, MNS1, BLM, MAP2K6
WHITFIELD_CELL_CYCLE_G2	-3.4	SRSF3, ZC3HC1, DEPDC1, RAD51C, YWHAH, NDE1, CDKN2D, HMGB3, CCSAP, DUSP4, MZT1, TOMM34, G2E3, ANP32B, SAPCD2, CKAP2, MIS18BP1, RRP1, HSPA1L, ARHGAP19, ANP32E, MDC1, CKS2, GAS2L3, KNSTRN, MCM4, TRIP13, NUP35, ECT2, SPDL1, HMMR, CENPE, POC1A, MKI67, TTK, BUB1, SPAG5, CCNB1, PRR11, CENPF, KIF20B, ANLN, CCNB2, GPSM2, CDC25B, TPX2, DLGAP5, SGO2, DEPDC1B, BUB1B, SHCBP1, AURKA, NUSAP1, KIF14, GTSE1, FOXM1, PBK, NUF2, CENPA, KIF2C, CCNA2, CEP55, CKS1B, BIRC5, PIMREG, CDCA3, PLK1, NEK2
REACTOME_TRANSLATION	-3.2	FAU, SRP72, MRPS35, RPN1, RPL29, RPL36AL, RPL23A, EIF2S3, MRPL27, EIF5B, RPL27, MRPS34, UBA52, MRPL40, AIMP1, EIF3F, SRP68, MRPL57, RPL11, EEF1G, RPL19, YARS2, RPS11, MRPL38, RPS18, RPS27, MRPS24, RPL18A, GADD45GIP1, MRPS14, MRPS16, RPL13, RPS14, MRPL35, MRPS27, EIF3G, RPS24, EIF1AX, MRPL30, MRPS22, EIF2B4, RPL37A, EEF1D, EIF2B2, RPS2, RPL18, MRPS18B, MRPS2, RPS26, MRPL55, MRPL9, RPS17, SSR2, MRPL34, EIF3H, EEF1E1, MRPL13, MRPL10, TRMT112, HARS2, MRPL3, MRPL44, RPL9, RPL22, MRPL37, SRP9, RPL30, MRPS21, RPS16, SPCS1, EEF2, MRPL15, RPLP2, RPS7, PTCD3, MRPL58, ERAL1, RPLP1, RPL26L1, RPL6, RPL39, EIF2S2, RPS4X, MRPL19, RPS27A, RPSA, RPL15, RPL7A, RPS20, MRPS12, FARSB, RPS19, IARS2, MRPL51, EIF3L, RPLP0, MTIF2, MRPS5, MRRF, RPL14, RPL21, MRPL22, RPL23, MRPL42, DARS2, MRPL45, MARS2, RPL32, RPS15A, EIF4B, RPL27A, RPS3, NARS2, RPL24, RPS25, MRPS30, EEF1A1, RPL36A, MRPL16, MRPS11, RPL26, RPL7, OXA1L, MRPL1, EIF2B3, FARS2, PABPC1, RPL4, SSR3, MRPL18, EIF3E, MRPL12, RPL13A, RPS29, RPL5, RPS12, RPS23, RPL12, RPL3, TARS2, MRPL24, MRPL39, RPL17, MRPS31, SEC11A, RPL22L1, MRPS23, RPL34, VARS2, FARSA, MRPS26, PPA2, MRPL47, EIF4A2, MRPL52, CARS2, RPS9, AARS2, RPS5, RPS6, EIF3B, RPL10A, MRPL4, MRPL2, MRPL17, RPL10, EEF1B2, EIF3M, MRPL46, RPS8, MRPS17, RPS3A, MRPL48, LARS2, MRPL11, MRPS9, EIF4EBP1, TSFM, MRPS6
REACTOME_SEPARATION_OF_SISTER_CHROMATIDS	-3.2	STAG1, CENPO, RCC2, NUP85, NUP107, RAD21, NDE1, NUP37, PPP2R5C, SMC3, SEH1L, BUB3, CENPL, CENPS, DSN1, CENPQ, SKA2, UBE2S, NUP160, KNTC1, TUBA1B, ITGB3BP, ESPL1, INCENP, SPDL1, ERCC6L, CENPI, CENPE, KNL1, BUB1, CENPF, CENPK, CDC20, SGO2, BUB1B, MAD2L1, NDC80, KIF18A, ZWILCH, CENPU, ZWINT, CDCA5, CENPM, SKA1, NUF2, CDCA8, CENPA, SGO1, SPC24, KIF2C, CENPH, SPC25, BIRC5, AURKB, UBE2C, PTTG1, PLK1
SCIAN_CELL_CYCLE_TARGETS_OF_TP53_AND_TP73_DN	-3.1	CCNF, CKS2, MCM6, DTYMK, CDK2, KIF23, CCNB1, CDC25B, TPX2, CDC20, MCM7, AURKA, CDKN3, GTSE1, CCNA2, CDC25C, UBE2C
REACTOME_MITOTIC_G1_PHASE_AND_G1_S_TRANSITION	-3.0	GMNN, RPA1, POLE3, CCNE2, CDC25A, WEE1, RBL1, POLA1, DBF4, CDK4, CDKN2A, MCM6, CDT1, MCM4, MCM5, CDK2, PCNA, FBXO5, POLE2, E2F2, CDC7, E2F1, MCM3, CDKN2C,

		RPA3, PRIM1, LIN9, MCM2, CCNB1, RRM2, TOP2A, ORC1, TK1, TYMS, MCM7, MCM10, CDC6, CDK1, ORC6, MYBL2, CCNA2, CDC45, CKS1B, POLA2, SKP2
WHITFIELD_CELL_CYCLE_G2_M	-3.0	SRSF3, ZC3HC1, DEPDC1, RAD51C, YWHAH, NDE1, CDKN2D, HMGB3, CCSAP, DUSP4, MZT1, TOMM34, G2E3, ANP32B, SAPCD2, CKAP2, MIS18BP1, RRP1, HSPA1L, ARHGAP19, ANP32E, MDC1, CKS2, GAS2L3, KNSTRN, MCM4, TRIP13, NUP35, ECT2, SPDL1, HMMR, CENPE, POC1A, MKI67, TTK, BUB1, SPAG5, CCNB1, PRR11, CENPF, KIF20B, ANLN, CCNB2, GPSM2, CDC25B, TPX2, DLGAP5, SGO2, DEPDC1B, BUB1B, SHCBP1, AURKA, NUSAP1, KIF14, GTSE1, FOXM1, PBK, NUF2, CENPA, KIF2C, CCNA2, CEP55, CKS1B, BIRC5, PIMREG, CDCA3, PLK1, NEK2
REACTOME_REGULATION_OF_TP53_ACTIVITY_THROUGH_PHOSPHORYLATION	-2.9	RPA1, RFC2, PRKAG1, MRE11, TAF4B, SSRP1, RFC5, DNA2, CDK2, CHEK1, RFC4, RPA3, BRIP1, RFC3, BRCA1, RMI2, RBBP8, TPX2, AURKA, EXO1, CHEK2, CCNA2, RAD9B, AURKB, BLM
REACTOME_EUKARYOTIC_TRANSLATION_INITIATION	-2.9	EIF4A1, EIF2B5, RPS13, FAU, RPL29, RPL36AL, RPL23A, EIF2S3, EIF5B, RPL27, UBA52, EIF3F, RPL11, RPL19, RPS11, RPS18, RPS27, RPL18A, RPL13, RPS14, EIF3G, RPS24, EIF1AX, EIF2B4, RPL37A, EIF2B2, RPS2, RPL18, RPS26, RPS17, EIF3H, RPL9, RPL22, RPL30, RPS16, RPLP2, RPS7, RPLP1, RPL26L1, RPL6, RPL39, EIF2S2, RPS4X, RPS27A, RPSA, RPL15, RPL7A, RPS20, RPS19, EIF3L, RPLP0, RPL14, RPL21, RPL23, RPL32, RPS15A, EIF4B, RPL27A, RPS3, RPL24, RPS25, RPL36A, RPL26, RPL7, EIF2B3, PABPC1, RPL4, EIF3E, RPL13A, RPS29, RPL5, RPS12, RPS23, RPL12, RPL3, RPL17, RPL22L1, RPL34, EIF4A2, RPS9, RPS5, RPS6, EIF3B, RPL10A, RPL10, EIF3M, RPS8, RPS3A, EIF4EBP1
REACTOME_APC_C_MEDIATED_DEGRADATION_OF_CELL_CYCLE_PROTEINS	-2.9	BUB3, UBE2S, CDK2, FBXO5, CCNB1, CDC20, BUB1B, MAD2L1, AURKA, CDK1, CCNA2, AURKB, UBE2C, PTTG1, PLK1, NEK2, SKP2
NAKAMURA_CANCER_MICROENVIRONMENT_DN	-2.8	PSMG1, PA2G4, DHFR, PSPH, SLC7A11, PSAT1, NDC1, RFC5, KNSTRN, TRIP13, RFC4, ASNS, CDCA2, BUB1, SPAG5, CCNB2, CDC20, AURKA, FAM83D, CDC6, KIF2C, FANCI
REACTOME_EUKARYOTIC_TRANSLATION_ELONGATION	-2.8	RPS13, FAU, RPL29, RPL36AL, RPL23A, RPL27, UBA52, RPL11, EEF1G, RPL19, RPS11, RPS18, RPS27, RPL18A, RPL13, RPS14, RPS24, RPL37A, EEF1D, RPS2, RPL18, RPS26, RPS17, RPL9, RPL22, RPL30, RPS16, EEF2, RPLP2, RPS7, RPLP1, RPL26L1, RPL6, RPL39, RPS4X, RPS27A, RPSA, RPL15, RPL7A, RPS20, RPS19, RPLP0, RPL14, RPL21, RPL23, RPL32, RPS15A, RPL27A, RPS3, RPL24, RPS25, EEF1A1, RPL36A, RPL26, RPL7, RPL4, RPL13A, RPS29, RPL5, RPS12, RPS23, RPL12, RPL3, RPL17, RPL22L1, RPL34, RPS9, RPS5, RPS6, RPL10A, RPL10, EEF1B2, RPS8, RPS3A
REACTOME_CYCLIN_A_B1_B2_ASSOCIATED_EVENTS_DURING_G2_M_TRANSITION	-2.7	CDC25A, WEE1, CDK2, CCNB1, CCNB2, CDC25B, PKMYT1, CDK1, FOXM1, CCNA2, CDC25C, PLK1
REACTOME_MITOCHONDRIAL_TRANSLATION	-2.7	MRPL27, MRPS34, MRPL40, MRPL57, MRPL38, MRPS24, GADD45GIP1, MRPS14, MRPS16, MRPL35, MRPS27, MRPL30, MRPS22, MRPS18B, MRPS2, MRPL55, MRPL9, MRPL34, MRPL13, MRPL10, MRPL3, MRPL44, MRPL37, MRPS21, MRPL15, PTCD3, MRPL58, ERAL1, MRPL19, MRPS12, MRPL51, MTIF2, MRPS5, MRRF, MRPL22, MRPL42, MRPL45, MRPS30, MRPL16, MRPS11, OXA1L, MRPL1, MRPL18, MRPL12, MRPL24, MRPL39, MRPS31, MRPS23, MRPS26, MRPL47, MRPL52, MRPL4, MRPL2, MRPL17, MRPL46, MRPS17, MRPL48, MRPL11, MRPS9, TSFM, MRPS6
BIOCARTA_ATRBRCA_PATHWAY	-2.7	MRE11, FANCC, FANCE, FANCD2, BRCA2, CHEK1, FANCA, BRCA1, FANCG, CHEK2, RAD51

REACTOME_G0_AND_EARLY_G1	-2.7	LIN54, TFDP1, MYC, CCNE2, CDC25A, RBL1, CDK2, PCNA, E2F1, LIN9, TOP2A, CDC6, CDK1, MYBL2, CCNA2
REACTOME_REGULATION_OF_TP53_ACTIVITY	-2.6	PPP2R5C, RPA1, RFC2, SMYD2, PRKAG1, MRE11, RBBP7, TAF4B, SSRP1, RFC5, CDKN2A, MAPKAP1, DNA2, CDK2, CHEK1, RFC4, RPA3, BRIP1, RFC3, BRCA1, RMI2, RBBP8, TPX2, AURKA, EXO1, CHEK2, CDK1, CCNA2, RAD9B, AURKB, BLM, MAP2K6
REACTOME_AURKA_ACTIVATION_BY_TPX2	-2.6	HAUS3, NEDD1, NDE1, CEP135, CDK5RAP2, TUBB, CEP57, HAUS7, CENPJ, HAUS6, CEP78, HAUS5, HAUS1, PLK4, CNTRL, HAUS4, HMMR, HAUS8, CEP152, TPX2, AURKA, CDK1, PLK1, NEK2
PID_AURORA_A_PATHWAY	-2.6	TACC3, BRCA1, CDC25B, TPX2, DLGAP5, AURKA, AJUBA, CENPA, BIRC5, AURKB
GEORGES_CELL_CYCLE_MIR192_TARGETS	-2.6	DEPDC1, GEN1, NEDD1, TENT4A, TTF2, RECQL, EFCAB11, KIAA1958, PSMC3IP, RAD21, OSBPL8, NDE1, CDKN2D, STAMBPL1, MSH6, ABCB10, UNG, FGF2, NOB1, TRIB3, CCSAP, NOXO1, PRADC1, GATB, SNRPD1, PHF6, ZNF165, TBC1D31, USP1, IQCC, MZT1, SLC7A11, PRIMPOL, NHLRC3, SLF1, FANCM, B4GALT6, CKLF, G2E3, TMEM200B, SLC39A8, CDC25A, RAD54B, SLC1A4, SPIN4, EPM2A, LMNB2, CD83, MIS18BP1, NSD2, CEP78, DSN1, UGT8, RFWD3, FBXO4, MT1X, CCDC15, NEMP1, HADH, SFR1, SLC16A7, ARHGAP19, ANG, DEK, CDCA4, CEP128, ATAD2, ATAD5, TEX30, MDC1, CDKN2A, STIL, GAS2L3, MCM6, RNASE4, SASS6, ASPM, JADE1, ASPH, SAMD13, CLSPN, PLK4, SMC4, C5orf34, EXTL2, CCDC18, TRIP13, FBXO5, MT1F, BRCA2, ECT2, CDC7, ZNF519, EME1, C1orf112, HMMR, ERCC6L, CENPI, CENPE, MCM3, FAM111B, SMC2, KIF23, RTKN2, C18orf54, RFC4, KNL1, PRIM1, MKI67, LIN9, BRIP1, HES6, E2F8, TICRR, TTK, BRCA1, ESCO2, SAP30, CIT, CENPF, KIF20A, KIF20B, ANLN, TROAP, DBF4B, ORC1, WDR76, CENPK, POLQ, HASPIN, GPSM2, LMNB1, TMPO, CDC20, DLGAP5, SGO2, KIF24, BUB1B, CLVS1, MAD2L1, PSRC1, KIF18A, CDKN3, NCAPH, FAM83D, MCM10, ARHGAP11A, CKAP2L, TRAIP, KIF14, GPR19, HJURP, RACGAP1, KIF15, RAD51, SKA1, KIFC1, DTL, NUF2, PIF1, CENPA, NEIL3, FEN1, RDM1, CEP55, POLA2, MNS1, BLM, NMU, FANCI
REACTOME_SWITCHING_OF_ORIGINS_TO_A_POST_REPLICATIVE_STATE	-2.5	GMNN, CCNE2, UBE2S, MCM6, CDT1, MCM4, MCM5, CDK2, MCM3, MCM2, ORC1, MCM7, CDC6, ORC6, CCNA2, UBE2C, SKP2
REACTOME_MITOTIC_G2_G2_M_PHASES	-2.5	BORA, NEDD1, NDE1, CEP135, CDK5RAP2, TUBB, CEP57, MZT2A, MZT1, HAUS7, CENPJ, CDC25A, WEE1, HAUS6, CEP78, HAUS5, HAUS1, PLK4, CNTRL, TUBA1B, CDK2, HAUS4, E2F1, HMMR, LIN9, FKBPL, HAUS8, CCNB1, CENPF, CEP152, CCNB2, CDC25B, TPX2, PKMYT1, AURKA, AJUBA, GTSE1, CDK1, FOXM1, MYBL2, CCNA2, CDC25C, PLK1, NEK2
REACTOME_E2F_MEDIATED_REGULATION_OF_DNA_REPLICATION	-2.4	TFDP1, MCM8, POLA1, E2F1, PRIM1, CCNB1, ORC1, CDK1, ORC6, POLA2
REACTOME_REGULATION_OF_PLK1_ACTIVITY_AT_G2_M_TRANSITION	-2.4	HAUS3, BORA, NEDD1, NDE1, CEP135, CDK5RAP2, TUBB, CEP57, HAUS7, CENPJ, HAUS6, CEP78, HAUS5, HAUS1, PLK4, CNTRL, HAUS4, HAUS8, CCNB1, CEP152, CCNB2, AURKA, AJUBA, CDK1, PLK1, NEK2
REACTOME_SUMOYLATION_OF_DNA_DAMAGE_RESPONSE_AND_REPAIR_PROTEINS	-2.4	NUP88, RING1, NUP155, RAE1, NUP62, NUP205, SUMO2, SMC1A, PARP1, STAG1, CBX2, SUMO3, NUP93, AAAS, NUP85, NUP107, RAD21, NUP37, SMC3, RPA1, SEH1L, CBX4, NDC1, MDC1, CDKN2A, NUP160, NUP35, BRCA1, BLM
WP_ATM_SIGNALING_PATHWAY	-2.3	ATF2, MRE11, CDC25A, FANCD2, MDC1, CDK2, CHEK1, BRCA1, CCNB1, CHEK2, CDK1, RAD51, CDC25C
REACTOME_KINESINS	-2.3	TUBA1B, CENPE, KIF23, KIF20A, KIF20B, KIF18B, KIF4A, KIF18A, KIF11, RACGAP1, KIF15, KIFC1, KIF2C

PID_ATM_PATHWAY	-2.3	ABL1, CTBP1, SMC1A, DCLRE1C, SMC3, MRE11, CDC25A, FANCD2, MDC1, BRCA1, RBBP8, CHEK2, CDC25C, BLM
BIOCARTA_G2_PATHWAY	-2.3	YWHAH, CDKN2D, CDC25A, WEE1, CHEK1, BRCA1, CCNB1, CDC25B, CHEK2, CDK1, CDC25C, PLK1
REACTOME_INITIATION_OF_NUCLEAR_ENVELOPE_NE_REFORMATION	-2.3	CCNB1, CCNB2, LMNB1, TMPO, VRK1, CDK1
REACTOME_TRANSCRIPTIONAL_REGULATION_BY_TP53	-2.2	PPP2R5C, RABGGTB, ATF2, G6PD, LAMTOR4, RPA1, RFC2, NELFA, SMYD2, PRKAG1, MRE11, CCNE2, RBBP7, CENPJ, GTF2H4, RBL1, POLR2E, TAF4B, FANCC, SSRP1, FANCD2, RFC5, MDC1, CDKN2A, MAPKAP1, DNA2, RRAGB, CDK2, PCNA, E2F1, CHEK1, RFC4, RPA3, E2F7, BRIP1, RFC3, CNOT7, E2F8, BRCA1, CCNB1, RMI2, RBBP8, TPX2, AURKA, EXO1, CHEK2, CDK1, CCNA2, RAD9B, CDC25C, BIRC5, AURKB, BLM, MAP2K6, FANCI
KENNY_CTNNB1_TARGETS_UP	-2.2	EBNA1BP2, HILPDA, TMEM94, EXOSC2, TFDP1, HAT1, SRSF7, SORBS1, CSE1L, CTSC, TACC3, TM4SF1, PLK4, ECT2, CCNB1, TK1, HMGB2
WP_MIRNA_REGULATION_OF_DNA_DAMAGE_RESPONSE	-2.2	MRE11, CCNE2, CDC25A, CDK4, FANCD2, CDK2, E2F1, CHEK1, BRCA1, CCNB1, CCNB2, MCM7, CHEK2, CDK1, RAD51, CDC25C, MIR17HG
REACTOME_PROCESSING_OF_CAPPED_INTRONLESS_PRE_MRNA	-2.2	SNRPB, NUDT21, SNRPE, SNRPG, CLP1, CSTF2T, CPSF1, NCBP2, CPSF2, PABPN1, CPSF3, SNRPF, LSM11, CSTF3, SLBP
REACTOME_DISEASES_OF_PROGRAMMED_CELL_DEATH	-2.1	RBBP7, CDC25A, DNMT1, POLA1, CDKN2A, EZH2, PRIM1, LMNB1, CDC25B, POLA2, CDC25C
REACTOME_TP53_REGULATES_TRANSCRIPTION_OF_CELL_CYCLE_GENES	-2.1	CCNE2, CENPJ, RBL1, CDK2, PCNA, E2F1, E2F7, CNOT7, E2F8, CCNB1, AURKA, CDK1, CCNA2, CDC25C
REACTOME_SUMOYLATION_OF_RNA_BINDING_PROTEINS	-2.1	NUP133, NUP88, RING1, NUP155, RAE1, HNRNPC, NUP62, NUP205, NOP58, SUMO2, CBX2, NUP93, AAAS, NUP85, NUP107, NUP37, SEH1L, CBX4, NDC1, NUP160, NUP35
REACTOME_DNA_DAMAGE_BYPASS	-2.1	USP1, RPA1, RFC2, POLE3, RAD18, RFC5, POLD3, PCNA, POLE2, POLD1, RFC4, RPA3, RFC3, PCLAF, DTL
REACTOME_MRNA_SPLICING_MINOR_PATHWAY	-2.0	SRSF6, ZCRB1, YBX1, DDX23, SNRNP35, SF3B2, POLR2G, SNRPB, SNRPE, SRSF1, SF3B3, SRSF2, SNRPG, NCBP2, GTF2F2, POLR2H, SNRPF, SRSF7, POLR2F, SNRPD1, POLR2E
